# High-resolution ecosystem changes pacing the millennial climate variability at the Middle to Upper Palaeolithic transition in NE-Italy

**DOI:** 10.1038/s41598-023-38081-1

**Published:** 2023-08-01

**Authors:** Federica Badino, Roberta Pini, Cesare Ravazzi, Milan Chytrý, Paolo Bertuletti, Eugenio Bortolini, Lydie Dudová, Marco Peresani, Matteo Romandini, Stefano Benazzi

**Affiliations:** 1 Department of Cultural Heritage, University of Bologna, 48121 Ravenna, Italy; 2 CNR-Institute of Environmental Geology and Geoengineering, Lab. of Palynology and Palaeoecology, Research Group on Vegetation, Climate and Human Stratigraphy, 20126 Milan, Italy; 3 Department of Botany and Zoology, Faculty of Science, Masaryk University, Brno, Czech Republic; 4 Department of Paleoecology, Institute of Botany, Czech Academy of Sciences, Brno, Czech Republic; 5 Department of Humanities, Prehistoric and Anthropology Sciences, University of Ferrara, 44100 Ferrara, Italy

**Keywords:** Climate-change ecology, Ecosystem ecology, Fire ecology, Forest ecology, Palaeoecology, Archaeology, Palaeontology

## Abstract

Observation of high-resolution terrestrial palaeoecological series can decipher relationships between past climatic transitions, their effects on ecosystems and wildfire cyclicity. Here we present a new radiocarbon dated record from Lake Fimon (NE-Italy) covering the 60–27 ka interval. Palynological, charcoal fragments and sediment lithology analysis were carried out at centennial to sub-centennial resolutions. Identification of the best modern analogues for MIS 3 ecosystems further enabled to thoroughly reconstruct structural changes in the vegetation through time. This series also represents an “off-site” reference record for chronologically well-constrained Palaeolithic sites documenting Neanderthal and *Homo sapiens* occupations within the same region. Neanderthals lived in a mosaic of grasslands and woodlands, composed of a mixture of boreal and broad-leaved temperate trees analogous to those of the modern Central-Eastern Europe, the Southern Urals and central-southern Siberia. Dry and other grassland types expanded steadily from 44 to 43 ka and peaked between 42 and 39 ka, i.e., about the same time when Sapiens reached this region. This vegetation, which finds very few reliable modern analogues in the adopted Eurasian calibration set, led to the expansion of ecosystems able to sustain large herds of herbivores. During 39–27 ka, the landscape was covered by steppe, desert-steppe and open dry boreal forests similar to those of the modern Altai-Sayan region. Both Neanderthal and Sapiens lived in contexts of expanded fire-prone ecosystems modulated by the high-frequency climatic cycles of MIS 3.

## Introduction

Marine isotope stage (MIS) 3 (∼ 60–30 ka), a period of intermediate global ice volume between MIS 4 and the Last Glacial Maximum (∼ MIS 2), was one of the periods of most unstable climate, closely interwoven with human evolution history. During MIS 3, climate variability was associated with abrupt atmospheric shifts over Greenland (Dansgaard–Oeschger [D–O] events), episodes of massive iceberg discharge and freshwater inputs into the North Atlantic up to the Iberian margin (Heinrich events [HEs])^1^, which generated climatic and ecological responses worldwide (Heinrich stadials [HS])^2^. Information about regional palaeoclimate during MIS 3 comes from isotope records of speleothems (e.g., Hölloch Cave, N-Alps^3^; Ascunsa Cave, S-Carpathians^4^, Pozzo Cucù Cave, Apulia, Southern Italy)^5^.

In Southern-Europe, along the Italian Peninsula (Monticchio, Castiglione, and Lagaccione, Italy)^6–8^ and in Mediterranean lowlands (Tenaghi Philippon and Megali Limni, Greece)^9–11^, temperate forests and forest-steppe were supported during Greenland Interstadials (GI), with steppe expansion during Greenland Stadials (GS), further exacerbated during HS^12,13^. Less extensive forest patches occurred in mountain areas during GSs (e.g., Ioannina, western Greece)^14^, where temperate tree population survival was favoured by orographic precipitation throughout MIS 3. Forests were also present in some parts of central and eastern Europe, *e*.*g.*, in river valleys, especially in the Carpathians^15^. In more continental areas, such as the lower Danube Plain and the semi-arid Pannonian Basin, vegetation formations became more open, likely due to drought stress^16^, and loess deposits formed repeatedly during MIS 3 (e.g., Nussloch and Willendorf sites)^17^. In western-central and western Europe, tundra vegetation prevailed during MIS 3 with relatively small stadial/interstadial fluctuations of tree and shrub populations: e.g., La Grande Pile, Les Echets, Bergsee (after 45 ka cal BP) and Füramoos pollen records^18^. Within this context, the replacement of Neanderthals by *Homo sapiens* is recorded across Europe in a diachronous and culturally articulated succession of local technocomplexes (e.g., Uluzzian), that replaced the Mousterian material cultures during the so called Middle to Upper Palaeolithic transition roughly between 45 and 40 ka cal BP^19^.

Relationships between climate fluctuations, environmental changes and the arrival of Sapiens at the expense of Neanderthals in Europe represent a hotly debated topic^5,20^.

Several factors contribute to the persisting uncertainty on the timing and mode of the earliest migration of Sapiens in Europe and the environmental context where this happened. In fact, most of the currently available information are inferred from chronological correlation with geographically remote records, i.e., Greenland ice cores^21^, rather than from regional environmental series. Moreover, only a subset of the several European archaeological sites recording the Middle to Upper Palaeolithic transition have been accurately investigated, or are well-documented and precisely dated^22,23^.

**Figure 1:**
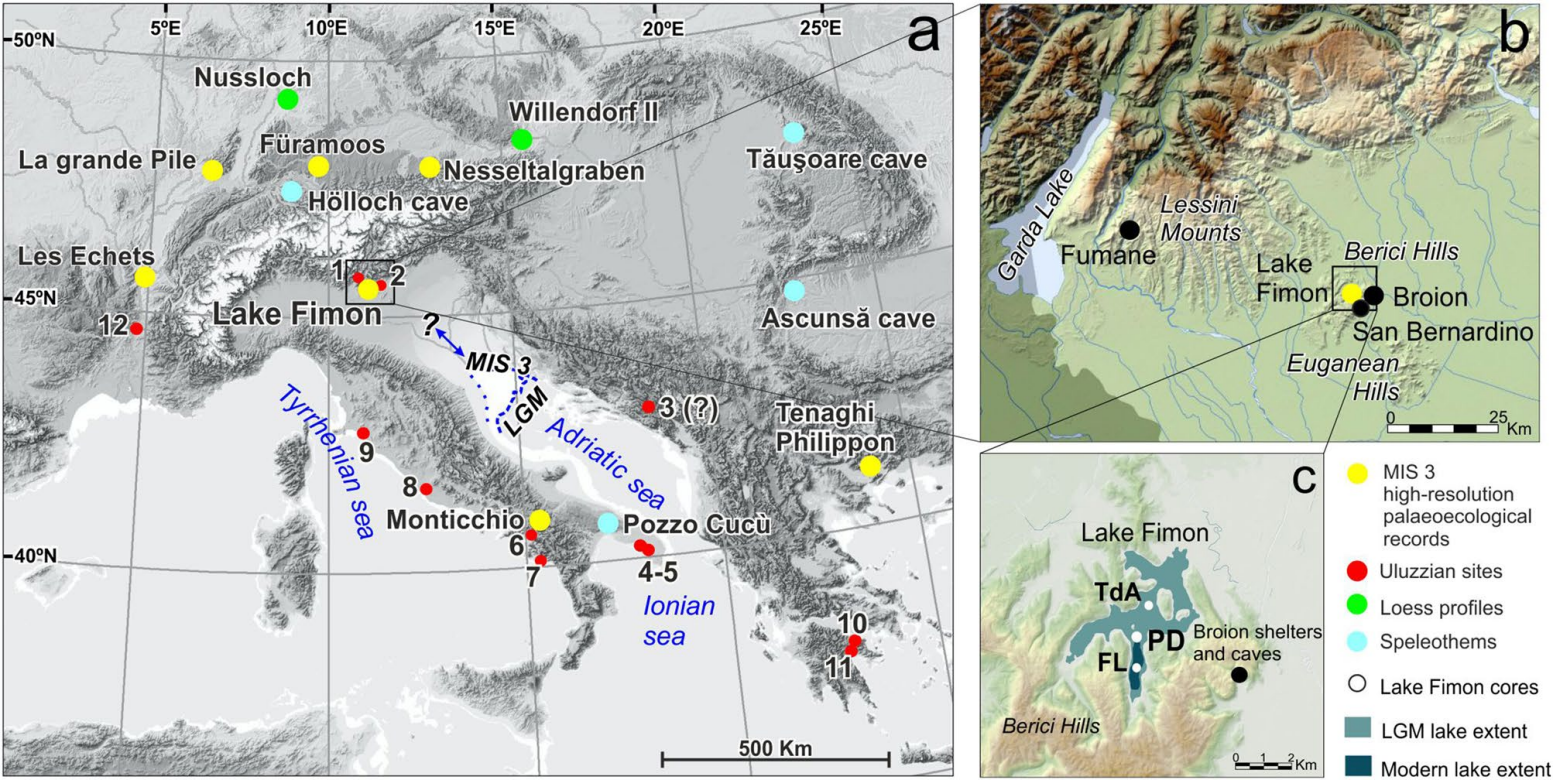
Geographical framework. (**a**) Location of high-resolution palaeoecological series entirely covering MIS 3 (yellow dots), speleothem series (light blue dots), loess profiles (light green dots) and Palaeolithic sites documenting the Uluzzian and Neronian cultures (red dots): (1) Fumane Cave; (2) Broion shelter; (3) Crvena Stijena; (4) Grotta del Cavallo; (5) Grotta di Uluzzo C; (6) Castelcivita; (7) Grotta della Cala; (8) Colle Rotondo; (9) Grotta La Fabbrica; (10) Klissoura Cave; (11) Kephalari Cave; (12) Mandrin Caves. Sea level has lowered by 120 m during the LGM^34^ and by 74 m during MIS 3 relative to the current sea level^35^ with new estimates suggesting higher sea levels between -18 and -40 m asl during MIS 3^36^. (**b**) The study area in NE Italy (Veneto region). Location of Lake Fimon and nearby Palaeolithic sites documenting the Uluzzian culture: Fumane cave (Lessini Mounts) and Broion shelter (Berici Hills), are highlighted: (**c**) Detail of the Lake Fimon area and of the coring sites (white dots): Ponte sulla Debba (PD), Torri di Arcugnano (TdA) and Fimon Lago (FL). Maps were created using ESRI ArcGIS 10.7 software (https://www.esri.com/software/arcgis) and the web page: https://www.freeworldmaps.net/europe/italy/veneto.html.

**Figure 2:**
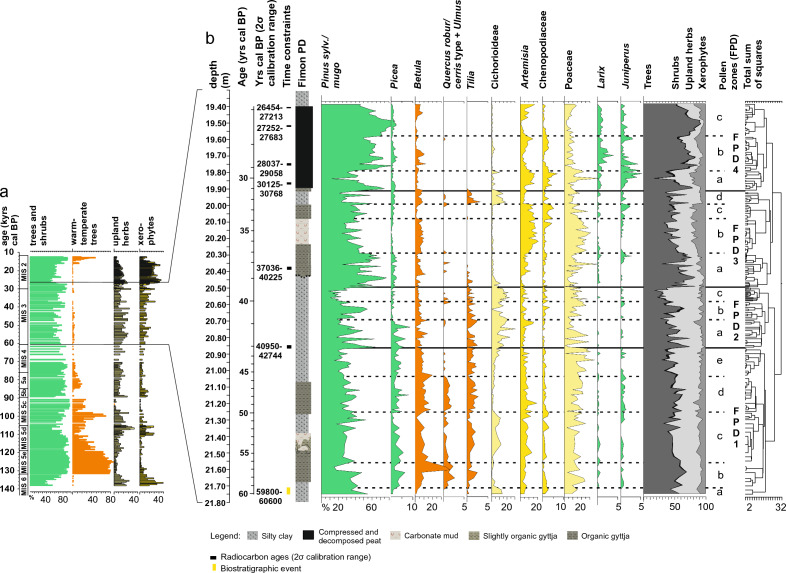
Summary of the palynostratigraphic data from Lake Fimon. (**a**) Synthetic composite pollen record obtained from cores FL (12–27 ka) and PD (> 27 ka) documenting the history of plant communities throughout the Late Pleistocene (modified after^30,37^). (**b**) New high-resolution synthetic palaeoecological record from the Fimon PD core for the period 60–27 ka cal BP: conifers (green), broad-leaved trees (orange), upland herbs (light yellow), xerophytes (yellow).

In the S–E fringe of the Italian Alps, in the sub-alpine area of the Berici Hills (S–E) and Lessini Mountains (N–W) (ca. 50 ka apart, Fig. 1b), favourable circumstances have fostered the accumulation and preservation of deposits documenting Neanderthals and Sapiens occupations at Fumane and Broion caves and shelters in the 45–40 ka time frame^24–29^. In the same area, the natural archive of Lake Fimon (Fig. 1) provides a palaeoecological series that entirely covers the last Glacial-Interglacial cycle^30–32^ (Fig. 2). Due to its favourable position and stratigraphic continuity, this sequence represents a suitable “off-site”^33^ reference record for NE-Italian Palaeolithic sites. In this paper, we present a new set of chronostratigraphic and high-resolution geochemical and microbotanical analyses carried out on the Fimon PD and TdA cores for the 60–27 ka interval (Figs. 1c and 2 and “Methods”). Specific objectives of this study are: (1) the reconstruction of millennial to sub-millennial environmental and fire regime dynamics throughout MIS 3, (2) the identification of the best modern analogues for MIS 3 ecosystems, and (3) the environmental contextualization of human occupation during the Middle to Upper Palaeolithic Transition in NE-Italy.

## Results

### Chronology

The Fimon PD record was age-depth modelled between 60.1 and 26.7 ka cal BP (mean ages) over a 2.36 m long core section (i.e., 21.75–19.39 m). We relied on ^14^C ages from two Lake Fimon sequences: Fimon PD and Fimon TdA cores, previously correlated for the Last Glacial-Interglacial cycle interval^37^. In this study, a targeted correlation for the MIS 3 interval was carried out relying on high-resolution litho-, chrono- and biostratigraphic data, allowing a precise arrangement of all the available Fimon TdA ^14^C dates along the Fimon PD record (see “Methods” section and Supplementary Fig. S1 for further details). The list of radiocarbon ages and the tie-point included in the model (Supplementary Fig. S3) is shown in Table 1. Deposits span ca. 33,300 years entirely covering Marine Isotope Stage 3. Chronological boundaries between pollen zones were identified at (95% probability): 43,096–41,079 cal BP (FPD1-2), 41,233–37,695 cal BP (FPD2-3), 33,885–30,098 cal BP (FPD3-4). The median resolution is 140 years for pollen and 80 years for sieved charcoal samples. Median accumulation rates are variable along the record: 0.006 cm/years for silty clay, gyttja and carbonate mud deposits (FL3-4) and 0.02 cm/years for peat interval (FL5), see Supplementary SI-1 and Supplementary Fig. S4 for further details.

**Table 1:** List of the radiocarbon ages and biostratigraphic events available from the Fimon PD and TdA sequences.

Radiocarbon ages from Lake Fimon—Ponte sulla debba (PD) and Torri di Arcugnano (TdA) cores								
Lab code	Core	Lithology	Original depth (m)	Fimon PD depth (m)	Material dated	^14^C Age BP	2σ calibration range (cal years BP) IntCal20	Median probability (cal years BP)
UBA-7831	Fimon PD	Peat	19.40–19.41	19.40–19.41	Bulk sediment	22,593 ± 115	26,816–27,211	26,953
UBA-7830	Fimon PD	Peat	19.51–19.52	19.51–19.52	Bulk sediment	23,165 ± 116	27,249–27,686	27,439
UBA-7829	Fimon PD	Peat	19.74–19.755	19.74–19.755	Bulk sediment	24,376 ± 187	28,014–29,056	28,591
UBA-15493	Fimon TdA	Peat	26.95–26.96	19.85–19.86	Bulk sediment	26,158 ± 97	30,125–30,768	30,376
UBA-46815	Fimon TdA	Slightly organic gyttja	27.66–27.68	20.37–20.39	Charcoal	33,990 ± 532	37,306–40,225	38,898
UBA-46816	Fimon TdA	Silty clay	28.61–28.63	20.84–20.86	Charcoal and wood	37,523 ± 837	40,950–42,744	41,952

Biostratigraphic events from Lake Fimon—Ponte sulla debba (PD) record								
Biostratigraphic event	Core		Original depth (m)	Fimon PD depth (m)	Isotopic correlated event		Age cal ka and error	
*Picea*—*Tilia* rise	Fimon PD	Silty clay	21.72–21.75	21.72–21.75	Start of GI 17		60.2 ± 0.4^30,38^	

### Gradients in pollen composition and dominant vegetation formations

**Figure 3:**
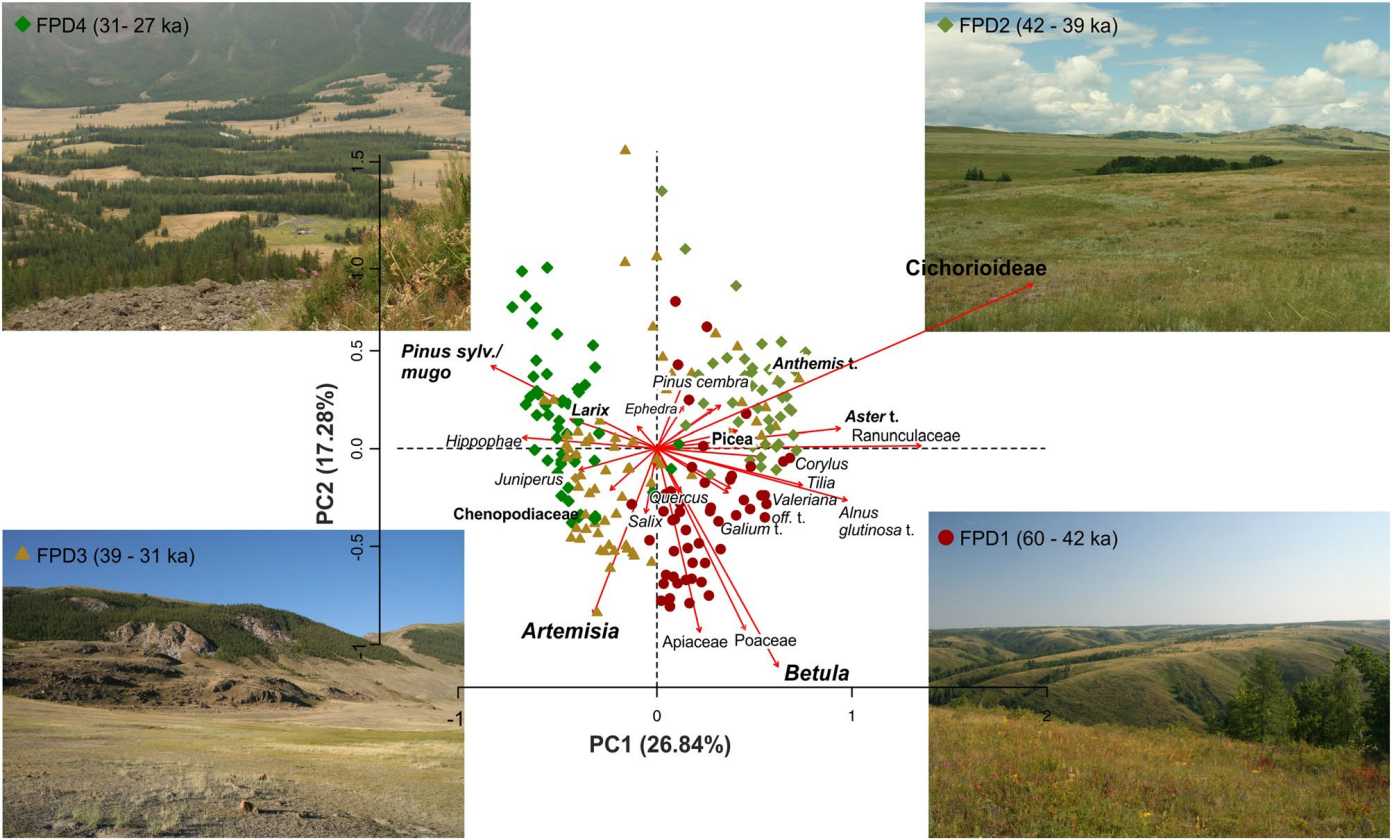
PCA ordination of terrestrial pollen taxa (> 2%) and sites showing changes in the pollen patterns in terrestrial ecosystems. Photos from the Southern Urals (FPD1 and FPD2) and the Altai Mountains (FPD3 and FPD4) show landscapes analogous to NE-Italy around Lake Fimon during the four time periods (pollen zones FPD1 to 4).

The Fimon PD fossil pollen record was analysed with a median time resolution of 140 years. Cluster analysis identified four pollen zones named FPD 1 to 4, the latter previously further subdivided into FPD 2 to 7^32^. Relatively high woody-pollen percentages (mean = 55%), dominated by *Pinus sylvestris/mugo* with peaks up to 93%*,* characterize the whole sequence (Fig. 2). Two major drops in forest cover occur in FPD2c and FPD4a pollen zones (Fig. 2). In the long term, land cover developed from a mosaic of grasslands and forest patches (FPD1), composed of a mixture of boreal and broad-leaved temperate trees, to dry boreal forests (FPD4) throughout more open phases dominated by herbs and xerophytes (FPD2-3). A notable change occurred at ca. 39 ka, i.e., the boundary between cluster groups FPD1-2 and FPD3-4, with samples showing positive and negative PCA1 scores, respectively (Figs. 2, 3).

The oldest pollen zone FPD1 is dominated by grasslands with patches of forests, including thermophilous *Ulmus* and *Quercus robur/cerris* type (up to 3%; Fig. 2), which expanded alongside *Picea,* whose percentages varied between 0.4 and 8% (Fig. 2)**.** Among other temperate tree taxa, *Tilia* persisted up to ca. 39 ka, while *Alnus glutinosa* type, *Corylus* and *Betula* are documented throughout the whole record, the latter showing rapid increases in relationship with enhanced fire frequency (Fig. 2 and Supplementary Fig. S7). Pollen zone FPD2 is mainly characterized by an expansion of Cichorioideae (up to 31%) and other Asteraceae (e.g., *Anthemis* type, *Aster* type, *Xeranthemum inapertum* type, Supplementary Fig. S3). Pollen zones FPD 3–4 are characterized by fluctuations between boreal forests with *Pinus sylvestris/mugo* and *Larix*, the latter with a continuous presence since 31 ka, and open environments dominated by *Artemisia* (mean = 8%) and Chenopodiaceae (mean = 1.5%; Fig. 2).

### Modern analogues

We used the MAT—Modern Analogue Technique to find the best-matching modern analogues for the Fimon PD fossil pollen assemblages. The adopted Eurasian calibration set consisted of 5978 modern pollen spectra: 5925 are from the Eurasian Modern Pollen Database vers. 2^39^ and 53 from the Southern Urals, collected in 2007 (see “Methods” for details). We also used climate-sensitive pollen indicators to better describe past floristic structures and ecoclimatic gradients (see “Methods” section).

Overall, 83% of the closest (first) modern analogues are considered “good analogues”, i.e., sqr. chord dist. < 5% (Supplementary Fig. S9, Supplementary Table S1)^40^. Modern analogues for fossil spectra included in pollen zone FPD1 (60–42 ka) belong to the “Central European mixed forests” (29%), the “Sarmatic mixed forests” (16%) and the “Kazakh forest steppe” (14%). Such environments are characterized by open mixed forests with a contribution of temperate broadleaved trees such as *Quercus, Tilia* and *Ulmus,* and a mosaic of grasslands with patches of *Pinus sylvestris* and deciduous trees. Other analogues belong to the “Sayan montane conifer forests” (20%) and the “Altai alpine meadow and tundra” (6%) ecoregions in semi-dry, mesic, wet and saline grasslands environments with both alpine and steppe species, and open larch forests. Species occurring frequently in the analogous Altai-Sayan grasslands and also found in NE Italy today include *Anthoxanthum alpinum*, *Aster alpinus*, *Bistorta major*, *B. vivipara*, *Galium verum* and *Rumex alpestris*, the moss *Rhytidium rugosum* and the lichen *Cetraria islandica*.

The 42–39 ka interval (pollen zone FPD2) is characterized by the marked expansion of Cichorioideae together with other Asteraceae (*Anthemis*, *Aster* type, *Xeranthemum inapertum* type), Ranunculaceae, *Geranium molle* type, *Ephedra fragilis* and *E. distachya* types and *Galium* (Supplementary Fig. S3). Such taxa, when combined, highlight a trend towards more open and dry conditions (see discussion). 48% of the pollen spectra from this pollen zone lack of good modern analogues; among the few statistically reliable modern analogues, several come from fallow lands, arid meadows and pasture habitats (Supplementary Table S1).

**Figure 4:**
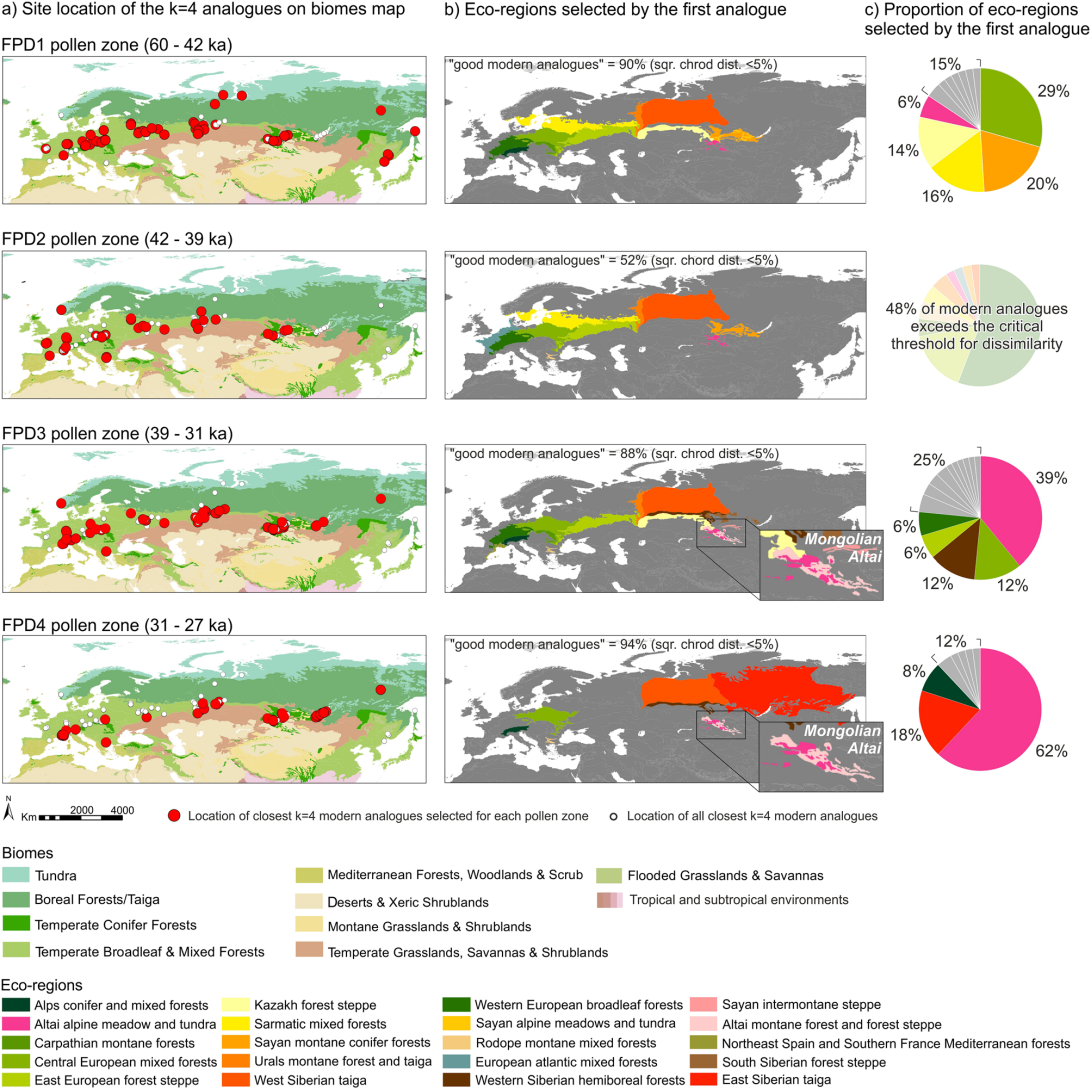
(**a**) Location of the four nearest modern analogues selected for each pollen zone: pollen zone FPD1 (60–42 ka cal BP); FPD2 (42–39 ka cal BP); FPD3 (39–31 ka cal BP); FPD4 (31–27 ka cal BP), plotted on Biomes map (RESOLVE Ecoregions 2017)^43^. (**b**) Eco-regions map^43^ shows the regions selected by the first modern analogue for each pollen zone. (**c**) Pie-charts showing the proportion of eco-regions selected by the first analogue for each pollen zone. The grey sections represent values < 5%. Maps were created using ESRI ArcGIS 10.7 software (https://www.esri.com/software/arcgis).

The modern analogues for the 39–31 ka interval (pollen zone FPD3) mostly belong to the “Altai alpine meadow and tundra” (39%), the “Central European mixed forests” (12%), the “Western Siberian hemiboreal forests” (12%), the “East European forest-steppe” (6%) and the “Western European broadleaf forests” (6%) ecoregions (Fig. 4). The best modern analogues in the Altai-Sayan region are again represented by a mosaic of various types of grasslands with woodland patches and tundra, the latter mainly at elevations of 2000–2200 m^41^. The Fimon PD fossil data are lacking or poorly representing tundra pollen indicators (e.g., *Dryas*, Ericaceae, *Salix*), suggesting a very little contribution of this habitat at Lake Fimon during MIS 3. Compared to the previous phases, dry steppe and desert-steppe sensu^42^ spread during pollen zone FPD 3 as documented by high *Artemisia* and Chenopodiaceae % values. Modern analogues include several species also found in northern Italy, e.g., *Allium strictum*, *Festuca valesiaca*, *Galium boreale* and *G. verum*, *Phleum phleoides*, *Stipa capillata* and *S. pennata*. Other analogues belong to taiga with *Larix*, *Picea* and *Pinus sibirica* (= *P. cembra* s. lat.), however, the latter species was always below 5% of total pollen in fossil spectra from Lake Fimon.

The youngest interval, i.e., 31–27 ka (pollen zone FPD4), finds its analogues especially in the “Altai alpine meadow and tundra” (62%), with a little contribution from the “East Siberian taiga” (18%) and the “Alps conifer and mixed forests” (8%) ecoregions (Fig. 4). The best modern analogues correspond to the same grassland, steppe and desert-steppe already detected for pollen zone FPD3, but several best analogues also come from forests, partly from *Larix-Picea-Pinus sibirica* taiga with *Vaccinium vitis-idaea* and abundant mosses, and partly from hemiboreal forests with *Pinus sylvestris*, *P. sibirica*, *Betula pendula* and a species-rich herb layer.

### Fire frequency, return interval, and severity reconstruction

We investigated changes in fire activity through the record of sieved charcoal particles (i.e., 62–125 μm, 125–500 μm and > 500 μm fractions) with a median time resolution of 80 years. Charcoal Accumulation Rates (CHAR) of particles > 125 μm at depths between 21.75 and 19.39 m were calculated (Supplementary Fig. S7). Signal to noise index (SNI) is greater than the critical value of 3 which indicates suitability for peak analysis^44^ for the 45% of the record (median = 2.7, min = 0 and max = 334.4; Supplementary Fig. S8). We avoided interpreting peaks corresponding to SNI < 3, mostly occurring in the lowermost part of the record (60–44 ka). Peak analysis revealed twenty-four statistically significant CHAR peaks showing variable magnitude between 0.0001 and 0.24 pieces cm^−2^ peak^−1^, with highest values between 31 and 27 ka. Fire return intervals range between 270 and 430 years and fire frequency up to 3 fires per 1000 years during phases of major biomass availability (GI 12, 8, 4 and 3 forest stages). No or low fire activity was registered during phases of prolonged biomass decrease and expansion of open vegetation (Supplementary Fig. S7).

## Discussion

### Vegetation dynamics and modern analogues for MIS 3 environments in NE-Italy

The best modern analogues for Fimon PD pollen spectra are mainly from the Eurasian temperate zone, especially from the transition zone between the Siberian boreal zone and the drylands of Central Asia. In particular, the Altai-Sayan area appears to be the best modern analogue ecoregion for most of the MIS 3 environments documented in the Fimon PD fossil record. This similarity has been already outlined for the Last Glacial ecosystems in Central Europe^15,45^.

**Figure 5:**
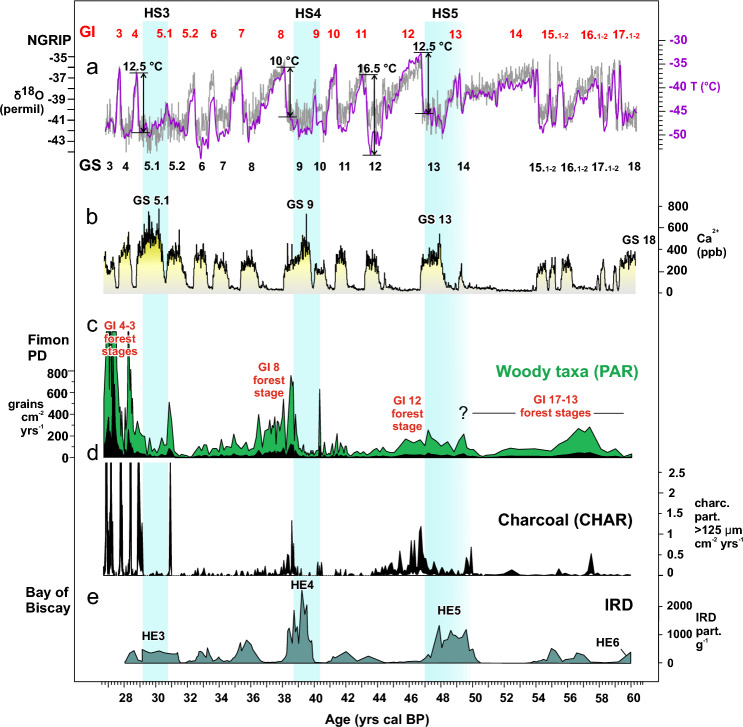
Summary plot showing (**a**) NGRIP δ^18^O record^47^ (grey curve), NGRIP temperature reconstruction^49^ (violet curve), and (**b**) NGRIP dust [Ca^2+^] record, plotted on the GICC05 chronology; (**c**) PAR (Pollen Accumulation Rates; grains cm^−2^ year^−1^) of Fimon PD woody taxa and (**d**) (CHAR) (Charcoal Accumulation rates; charcoal fragments > 125 µm cm^−2^ year^−1^) of Fimon PD charcoal plotted on the Fimon chronology (see “Methods” and Supplementary Fig. S4); (**e**) Ice-Rafted Debris (IRD) record from the Bay of Biscay: sediment core MD04-2845; Sanchez Goñi et al., 2008; age model < 31.8 ka cal BP according to Ref.^50^. Grey bands indicate Heinrich Stadials: HS5 (50/49–47.3 ka)^2,48^; HS4 (40.2–38.3 ka)^51^, HS3 (30.4–29.1 ka)^52^.

Although the landscape around Fimon was a mosaic of meadows, steppe and open forests throughout MIS 3, it changed over time, particularly in the proportion and composition of forest patches and the ratio of wet/mesic to dry grasslands. During the 60–42 ka interval (FPD1), several modern analogues suggest that the landscape may have been similar to the modern Eastern European forest-steppe such as that in the Southern Urals, where temperate broadleaved trees (*Quercus, Tilia, Ulmus*) are mixed with boreal trees^46^. These environments likely expanded during millennial interstadial phases between 59 and 44 ka: GI16, GI14 and GI12^47^, although the analogues for GI12 forest stage (47–44 ka) are both from Central European and Altai-Sayan regions which may suggest an increase in climatic continentality (Supplementary Table S1). In this framework the expression of Heinrich Stadial 5, i.e., 50/49–47.3 ka^2,48^, is possibly related to a phase of woody biomass decrease with most of the modern analogues recognized in the Sayan montane area (Fig. 5 and Supplementary Table S1). However, the coarser sample resolution and chronological control of this phase in our record prevents its conclusive attribution and correlation.

48% of pollen spectra between 42 and 39 ka (FPD2) finds no analogues among modern Eurasian pollen spectra (see methods; Fig. 4 and Supplementary Table S1), likely because of the very limited number of modern sites with Cichorioideae values as high as 10–30% (only 31 sites out of 5925 modern pollen assemblages). Although this condition prevents any intepretation in terms of modern analogue vegetation structure, some considerations can be made on the basis of fossil pollen data. Fimon PD pollen assemblages share similarities with steppe forb/shrub Plant Functional Type (PFT) described in Ref.^53^. Several indicator taxa (e.g., Asteraceae such as *Xeranthemum inapertum* type, *Centaurea scabiosa* and *C. nigra, Anthemis type*) together with Cichorioideae and Rubiaceae*,* roughly recall the set of late-flowering herbs blooming today in eastern European meadow steppe (e.g., *Galium verum*, *Centaurea ruthenica*, *Jurinea linearifolia*, *Serratula xeranthemoides*, *Linosyris villosa*)^54^. The scarcity of pollen surface samples in the EMPD2 dataset from the ecozone stretching between Ukraine and Southern Urals may have influenced the MAT results. A step towards drier condition occurred between 40.2 and 38.7 ka: arboreal pollen sank to values < 30%, *Pinus cembra* patches replaced *Pinus sylvestris/mugo* woodlands and xerophytes (*Ephedra* and *Hippophaë*) expanded with *Galium* and *Anthemis* type (Supplementary Fig. S3) indicating sparsely vegetated areas probably exposed to wind erosion and dust deflation. This chronological phase corresponds to HS4 (40.2–38.3 ka)^51^.

Between 39 and 27 ka (FPD3-4), the landscape was more diverse than the 60–39 ka interval. It included all types of grasslands, from wet to dry, up to desert-steppe (sensu^42^) with *Artemisia* and Chenopodiaceae semi-shrubs which are more drought-resistant than grasses, and today replacing them in the transitional region between steppe and semi-desert^54^. The interpretation of an open landscape seems to be in conflict with a relatively large proportion of arboreal pollen, which is over 40% for most of this interval. However, this is largely represented by *Pinus sylvestris/mugo* type, a wind-pollinated conifer which produces extremely high amounts of pollen that can be transported over long distances. Surface pollen spectra in the modern steppe-tundra landscape of Central Asia contain a large proportion of *Pinus* pollen, although *Pinus* is rare or absent from the surrounding landscape^41^. These open formations mainly expanded during GS8 to 4 and particularly during HS 3 (30.4 and 29.1 ka)^52^ (Fig. 5). Today such vegetations composed of xerophytes or mesoxerophytes are widespread from the Ural Mountains to the Mongolian Altai in continental, temperate to temperate-cold (subarctic/subalpine) climates where annual precipitation ranges from 400 mm in the north to 200 mm in the south^55,56^. During interstadials, boreal forests expanded and their tree layer consisted mainly of *Pinus* (*P. sylvestris* and *P. mugo*, although *P. cembra* could also have been present locally) and *Larix* mostly since 31 ka (Fig. 2). Despite the relatively low proportion of *Larix* in pollen spectra, it may have been the dominant tree in most of these forests, as this species is highly underrepresented in pollen spectra due to its low pollen productivity^57^ and limited dispersal. This forest-steppe ecogradient is today characterised by a continental timberline limit (300 mm annual rainfall and boreal continental climate)^58^ with a dry, but not drought period in mid to late summer^42^. The main difference between pollen zones FPD3 and FPD4 is the larger proportion of forests in the latter, pointing to more humid climate conditions^42^. Considering the landscape pattern in the modern Altai-Sayan region and adjacent areas, it is likely that forests were mainly occurring at sites with a more stable moisture supply, i.e., near streams, valley floors and on north-facing slopes^59^.

### Fire signal modulation throughout MIS3

The high-resolution sieved charcoal record from Fimon PD highlights enhanced fire activity during/at the boundary of interstadial forest stages, especially those following Heinrich stadials. Most of the local fires are registered during millennia-long interstadials (e.g., GI8 and GI12), while shorter interstadials have a weaker fire signal with the exception of GI4 and GI3 forest stages (see below). Surprisingly, the 60–50 ka interval, also containing long interstadials (e.g., GI16 and 14), diverges from this pattern, registering subdued fire activity (Fig. 5).

Fire activity mirrored the woody biomass rise and decline during millennia-long forest stages (Fig. 5). For example, at the GI8 onset, a sharp rise of woody pollen accumulation rates (from 25 to 120 grains cm^−2^ years^−1^) and charcoal influx (from 0.03 to 0.1 particles cm^−2^ years^−1^) is synchronous at the stratigraphic resolution of the record (i.e., 70 years) between 38,780 and 38,710 years cal BP (Fimon PD modelled mean ages). The adopted resolution matches the duration of stadial-interstadial transitions, estimated to be about 100–200 years^60^, suggesting that local fires occurred concurrently with the warming-induced biomass rise following Heinrich stadials. A different fire signal modulation is recorded during GI4 and GI3, when charcoal peaks are documented at the onset and at the end of interstadial phases, rather than extending to the overall phases of high forest biomass, as observed instead in GI12 and GI8. A different structure in vegetation and in climate transitions between interstadials may account for the observed changes in fire regimes. For example, in the Fimon pollen record boreal woodlands spread during GI4 and GI3, and climate transitions displayed extreme amplitude shifts, up to 12.5 °C in Greenland^49^. The high magnitude signal of GI4 and GI3 fire episodes in the Fimon record may have been influenced by the establishment of a fire-prone palustrine area (ca. 5.7 km^2^) for 3.8 ka, i.e., between ca. 31 and 27 ka^32^. Indeed, since bogs are more vulnerable to burn in early season than other ecosystems, such local conditions may arguably enhanced fuel consumption per fire episode, in turn favouring extensive fires^61^. Also, peat deposits are more adapted archives for the registration of individual fire events than lake sediments^62^.

**Figure 6:**
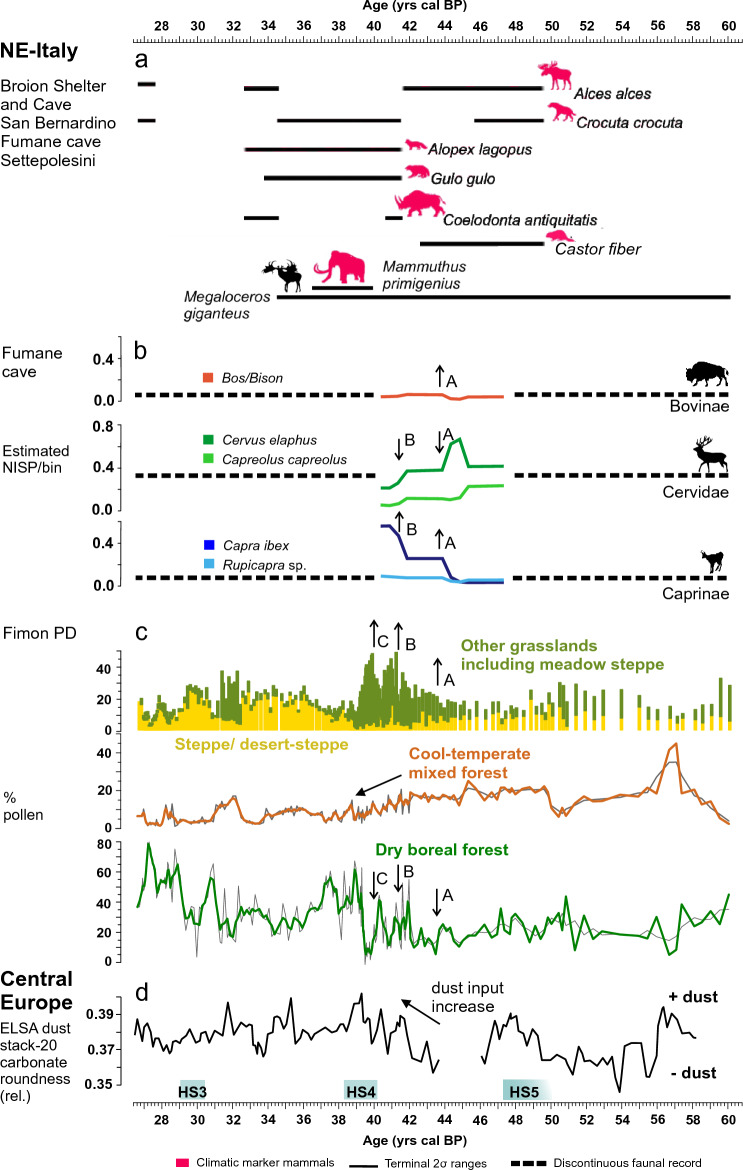
Eco-climatic proxies (from top to bottom): (**a**) zoological data from the Broion shelter-cave and the San Bernardino Cave for the Berici Hills; from the Fumane cave for the Lessini Mountains^63^; from Settepolesini for the Po Plain ^64^; (**b**) Estimated diachronic relative frequency of selected (diagnostic) ungulate taxa based on aoristic sums of NISP data computed for 500-year temporal bins in the interval 47.5–40 cal BP^63^. Dashed lines indicate discontinuous presence of the same taxa before and after the period with quantitative data; (**c**) Synthetic pollen curves including taxa grouped according to their ecology and climatic preferences, also highlighted by multivariate and correlation analyses (Fig. 3, Supplementary Figs. S5 and S6): Steppe/desert-steppe (*Artemisia*, Chenopodiaceae, *Hippophaë*); Other grasslands including meadow steppe (Cichorioideae, *Anthemis* type, *Aster* type, *Xeranthemum inapertum* type, Ranunculaceae, *Geranium molle* type, *Ephedra fragilis* and *Ephedra distachya* types); Cool-temperate mixed forest (*Alnus glutinosa* type, *Betula*, *Quercus robur/cerris* type, *Tilia*, *Picea*); Dry boreal forest (*Pinus sylvestris/mugo*, *Larix*, *Juniperus*); (**d**) Central Europe: ELSA Dust Stack-20 terrestrial sediment core, carbonate grain roundness as regional dust proxy^65^. Light blue rectangles indicate Heinrich Stadials: HS5 (47.3–49/50 ka)^2,48^; HS4 (40.2–38.3 ka)^51^, HS3 (29.1–30.4 ka)^52^.

The increase of fire activity during interstadials throughout the Last Glacial Period has already been documented in long marine and terrestrial records^50,66^. Unfortunately, the low time-resolution of most of the available charcoal records for MIS 3 limits their comparison at a centennial to decadal scale.

Overall, data from Fimon PD record indicate that fire regime in NE-Italy during MIS 3 was mainly driven by changes in biomass, in turn modulated by high-frequency climatic cycles.

### Palaeoclimatic and palaeoecological dynamics throughout MIS 3 in NE-Italy

**Figure 7:**
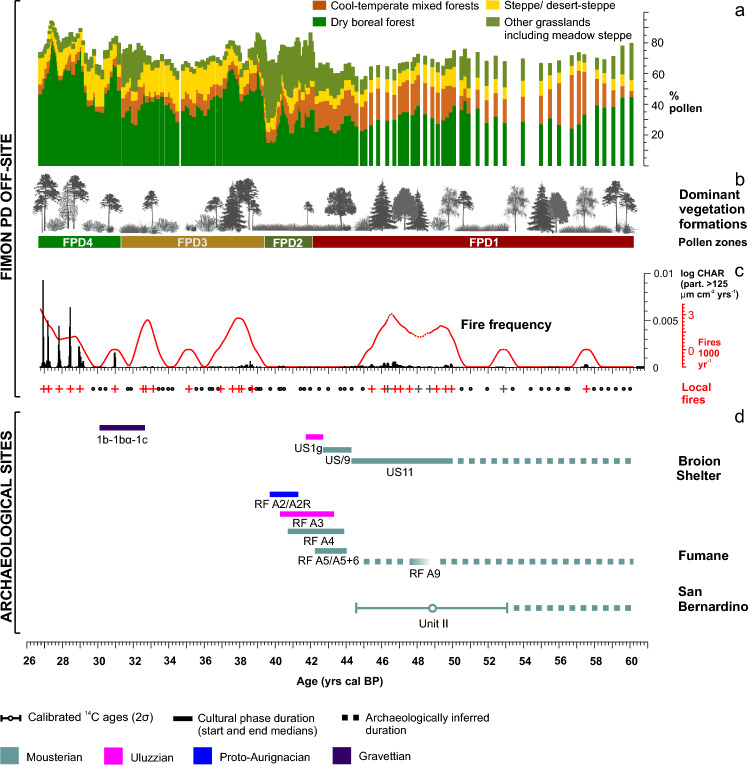
Comparison between environmental conditions reconstructed from the off-site Fimon PD palaeoecological record covering the 60–27 ka interval and cultural proxies from selected archaeological sites (NE-Italy). The records are plotted on their own time scale. Key to the panels: (**a**) Fimon PD synthetic pollen record; (**b**) Sketch of the changes in dominant vegetation formations through time; (**c**) Fimon PD interpolated charcoal accumulation rates (pieces cm^−2^ year^−1^) (black histograms), fire frequency (red line) and statistically significant local fire events plotted as red “ + ”symbols; (**d**) Broion shelter, San Bernardino cave (Berici Hills) and Fumane cave (Lessini Mountains) cultural and chronological sequences^27,69–71^.

In this section the Fimon PD palaeoecological data are discussed in the light of climatic forcing and integrated with previous studies on small and large mammals, and bird series from archaeological records (Fumane cave, San Bernardino cave and Broion shelter) and open-air contexts in NE-Italy (Settepolesini) during MIS 3. Long interstadials characterized the first part of MIS 3: GI16.1 (58–56.5 ka), GI14 (54.2–49.6 ka) and GI12 (46.8–44.2 ka) (NGRIP GICC05 chronology), as indicated by isotopic records in the eastern Mediterranean and Europe^3^. Open cool-temperate mixed woodlands expanded in NE-Italy (Fig. 6) similarly to what documented in other S-European pollen records^12^. The presence of open woodlands is also documented by small mammal assemblages uncovered in Mousterian units of the Fumane cave: A11 to A4 dated to 56–44 ka^67^ (Fig. 7); and micromammal associations of Unit II at San Bernardino where *Microtus agrestis* and *Apodemus gr. sylvaticus*-*flavicollis* are the most abundant species^68^. Forests likely experienced a contraction during HS5, however, this interval is not adequately resolved in the Fimon PD sequence (Fig. 5), but this contraction is known from other S-European and Mediterranean high-resolution pollen records^12^. Data from small mammal assemblages in the Mousterian units A7 and A6 older than 45 ka of Fumane cave, support a decrease in woodlands possibly related to HS5^67^. Studies on large mammals in cave and shelters of the Berici Hills (Supplementary SI-2) indicate a prevalence of cervids (e.g., *Cervus elaphus*, *Capreolus capreolus, Megaloceros giganteus*) over caprids (*Capra ibex* and *Rupicapra rupicapra*) and bovids (*Bison priscus* and *Bos primigenius*)^63,69^ and Supplementary SI-3. Estimated relative frequencies trends of change of ungulates between 47 and 41 ka in the Fumane cave show that *Cervus elaphus* reached its maximum between 45 and 44 ka during a phase of cool mixed forests expansion in the Fimon PD record during GI 12 (Fig. 6).

After GI12, a major cooling is centred on GS12 (44.3–43.3 ka)^47^ as documented in NGRIP T reconstruction^49^ (Fig. 5a). Despite this stadial is less prominent in the Atlantic record^72^, its expression is well recognizable over Europe as an extremely cold phase likely occurring over Western Europe, evidenced by deep frost figures in the loess/palaeosol records from northern France at ∼ 44–43 ka cal BP^73^ and Central Europe^74^. In the northern Alps, glaciers advanced between 46.3 and 42.9 ka cal BP and tundra became dominant since 43 ka (Nesseltalgraben, South-eastern Germany^75,76^). Increasing dust input is recorded in the ELSA dust stack since 44 ka, peaking at ca. 39 ka during HS4^65^ (Fig. 6). The Fimon PD record documents a major decrease in dry boreal forests with grasslands steadily increasing at ca. 44 ka (onset of GS 12, “A” event—Fig. 6). At ca. 42 ka grasslands expanded at the expense of cool-temperate mixed forests (“B” event—Fig. 6) with dry habitats and sparsely vegetated areas peaking at HS4 (“C” event—Fig. 6). These stepped events recalled the main changes in the estimated relative frequencies of ungulates at Fumane cave (Fig. 6b). The abundance of *Cervus elaphus* and *Capreolus capreolus* started to decline at ca. 44 (“A” event in Fig. 6) in favour of *Capra ibex* and *Rupicapra* sp. and further at ca. 42 ka (“B” event in Fig. 6). In the same time interval *Bos* and *Bison* show a quick unimodal trend^63^. A cold and dry period was indicated by small mammal assemblages in units A3 to A1 (Fumane cave), dated to between 44 and 38.9 ka cal. BP^27,67^. Alpine ibex, chamois, and marmot were favoured at low altitudes, as well as arctic fox and wolverine. The Po Plain was, in contrast, inhabited by woolly rhinoceros, mammoth, giant deer and bison (Fig. 6 and Supplementary SI-3)^63,64^.

GI8 is recognizable as a prominent interstadial phase, marked by a major boreal forest expansion in the Fimon PD record (Figs. 2 and 5), which is also observed in most of central and southern-European high-resolution records^12,18,77^ and possibly indicated by small mammal assemblages in units D1c and D1d in the Fumane cave^67^. From GS8 onwards (i.e., since 36.5 ka) NGRIP [Ca^2+^] record indicates increasing dust input frequency in Greenland^47^ (Fig. 5) and major dustiness peaks over Europe has been recorded at 35 and 32 ka (ELSA dust stack^65^), possibly correlated to two major contractions in dry boreal forests at the Fimon site (Fig. 6). Overall, between ca 39–31 ka, in a context of steppe and desert-steppe expansion, cervids declined sharply, while ibex and chamois reached their peak (see Fumane cave)^63^. In addition to the persistence of the wolverine, all mustelids, wolves and felids (*Panthera leo*, *Panthera pardus*, *Lynx lynx*) show a general increase, accompanied by hyena (*Crocuta crocuta*). Mammoth and giant deer persisted in the Po Plain^64^.

The most recent interval documented in the Fimon PD record, from 31 to 27 ka, is characterized by the occurrence of carnivores (*e.g*., cave bear), herbivores like moose (*Alces alces*), already documented in previous phases, and also fish and water birds in the cave and shelters of Berici Hills^78^ (Supplementary SI-3). This phase is marked by the GS 5.1 (HS 3) oscillation which produced a major steppe expansion, as documented elsewhere in the southern Alpine foreland (Casaletto Ceredano site^79^), while the short and pronounced interstadials following HS3 led to the renewed expansion of dry boreal forest up to 27.3 ka^31,32^. The HS3 event is also inferred based on small-mammal remains at the Fumane cave (unit D1e, with no cultural remains) and other sites in Italy, such as Riparo Mochi (first three phases of unit D), Grotta Paglicci (layers 22c-b)^80^ and Broion cave (layers G2-F)^81^. A coeval palaeobotanical record from the open-air site of Piovesello (watershed of the Northern Apennine ^82^) also speaks for an arid rocky landscape above the timberline.

### Placing the Middle to Upper Palaeolithic transition in an environmental context

The Fimon PD palaeoecological record provides the unique opportunity to discuss the environmental context where last Neanderthals and early Sapiens lived during MIS 3 in NE-Italy. Cultural changes across the Middle to Upper Palaeolithic transition have been acquired from available archaeological sites: three of them were selected due to their extensive radiocarbon chronology and cultural data, namely the Broion shelter, and the Fumane and San Bernardino caves (Figs. 1b, 7).

Neanderthals inhabited NE-Italy in a mosaic of grasslands and forest patches, composed of a mixture of boreal and broad-leaved temperate trees as those occurring today in the Southern Urals, Central-Eastern Europe and central-southern Siberia (Fig. 3). These ecosystems supported cervid populations (e.g., *Cervus elaphus, Capreolus capreolus, Megaloceros giganteus*), which prevailed over caprids and bovids as documented in Late Mousterian units at cave and shelters of Berici Hills^63^. Neanderthals likely dealt with natural fires, e.g., up to 3 local fire events per 1000 years, that could enclose one or more fires each, between 47 and 44 ka (GI12 forest stage; Figs. 5 and 7). In the eastern slope of the Berici Hills close to Lake Fimon, very late Middle Palaeolithic contexts document fireplaces with abundant bones, burnt flints and sparse charcoal particles at Grotta di San Bernardino^83^ and at the Broion shelter^28^. Detailed anthracological investigations carried out at the De Nadale cave suggests that, at the onset of MIS 4, Neanderthals preferentially exploited spruce/larch together with pine (*Pinus* tp. *sylvestris*) and birch^84^. A preferential exploitation of larch is also documented in charcoal assemblages and primary combustion features at the Fumane cave (Lessini Mountains; units A5–A6 and A9)^85,86^, 50 km away from Lake Fimon. The recurrent selection and use of this wood seems to disagree with the sporadic presence/low abundance of larch in the Fimon PD record (Fig. 2). However, we can relate this bias to the underrepresentation of larch in the regional pollen rain (see above). Indeed, this boreal species likely played an important role in the forests at the NE Alpine border, especially at the continental and upper alpine timberlines^31^.

Sapiens at Fumane (Unit A3) and at the Broion shelter (US1g), attested since 44–43 ka BP, experienced an initial phase of increasing woodland opening and grasslands expansion peaking during HS4 (Figs. 6, 7). Between ca. 44–38 ka, climate conditions have been dry enough to promote fires, but that the high degree of landscape openness resulted in very limited or absent local fuel availability for fire to spread (Fig. 7). This vegetation, which finds very few good modern Eurasian analogues, led to the expansion of ecosystems able to sustain large herds of herbivores, particularly Ibex, chamois and large mammals, and their hunters in the Great Adriatic-Po Region (GAPR). The occurrence of woolly rhino (*Coelodonta antiquitatis*), wolverine (*Gulo gulo*) and arctic fox (*Alopex* cfr. *lagopus*) as climatic markers in Uluzzian units further supports this picture. Other hints in this direction come from the Broion cave pollen record from units containing Upper Palaeolithic industries (“He” unit onwards)^87^ younger than ca. 52–42 ka (approximate time range inferred from two old conventional radiocarbon dates from the “I” unit)^88^. Pollen data show a major environmental change marked by the expansion of Cichorioideae-dominated grasslands comparable to findings from the Fimon PD and TdA records (Supplementary Fig. S1). Although it’s well known that pollen deposition in caves is complex and subjected to stochastic elements^89^, a suitable correlation with an “off-site” record corroborates the consistency of the Broion cave record that preserved the signal of main environmental transformations at a millennial time scale. Similar findings were observed north of the Alps, where early Sapiens lived in a cold steppe-type environment expanding since 43.5 ka (Willendorf II)^74^. The prominent GS 12 stadial phase (44.3–43.3 ka, Fig. 5) may have played a role in triggering these dynamics in S-European ecosystems^4^, although its expression is less remarkable in the Mediterranean^90^. After the Uluzzian phase, the Proto-Aurignacian one developed in a context of extremely open and harsh conditions (Fig. 7) roughly corresponding to GS 10-9/HS4, which favoured hunting of alpine ibex and chamois^91^. The following Gravettian cultural phase documented at the caves and shelter of the Broion karstic complex between ca. 32.6 and 30.1 ka, is established in widespread steppe/forest-steppe environments, the latter characterized by fire-prone boreal ecosystems^32,92^, which finds most of their best modern analogues in the Altai-Sayan region (Figs. 4 and 7). Gravettian hunters, who settled the northern edge of the GAPR, were fully adapted to this ecological context. A long-range mobility through this wide alluvial plain, e.g., between sites located over 250 km apart, is attested by petroarchaeological evidence at both italian and balkan sides^93^.

The long-term perspective offered by the present work suggests that Neanderthal and Sapiens locally developed different adaptive strategies to cope with marked changes in their ecosystems and—possibly—with variable demographic pressure over time. Evidence presented here pave the ground for future investigations on the relationship between resource management, technology, and palaeoenvironmental dynamics in NE-Italy.

## Methods

### Chronology

The Fimon Ponte sulla Debba core (acronym: Fimon PD) chronology was developed for the 21.75–19.39-m interval (Supplementary Fig. S4). We used six ^14^C dates made on bulk peat and terrestrial macroremains (wood and charcoal fragments) and one biostratigraphic tie point^30^. Three radiocarbon dates were obtained from the Fimon PD core and three others from the Fimon Torri di Arcugnano (TdA) core (Table 1). All ages were computed in a single age-depth model after litho-, bio- and chronostratigraphic correlation of the two sequences (see Supplementary Fig. S1 and Ref.^37^. ^14^C dates made on pollen concentrates and bulk samples from the Fimon PD interval between 19.885 and 21.75 m were excluded from our modelling since they mostly yielded age reversals, as previously demonstrated^30,32^; Supplementary Fig. S1). The tie-point identified at 21.72 m was obtained by tuning the marked rise in *Tilia* and *Picea* pollen curves with the GI 17 onset (i.e., 60.2 ± 0.4 ka^38^, see Ref.^30^ for further details). This is in agreement with the well-established evidence of a significant forest recovery at the end of HE 6 in Mediterranean pollen records^94,95^ as well as at Azzano Decimo^96^. Radiocarbon dates were calibrated using the IntCal20 calibration curve^97^ and the age model was calculated using OxCal 4.4 software^98^. We used standard codes and commands in OxCal, including Poisson-Process modelling with variable *k* parameter^99^. Precision was calculated as 95% confidence ranges for the age estimates of each depth (interpolation rate of 0.5 cm^−1^).

### Loss on ignition (LOI)

Loss-On-Ignition was performed on 367 volumetric samples from Fimon PD and 178 volumetric samples from Fimon TdA with an automated LECO TGA 601 thermo-gravimetric analyzer (LECO Corporation, USA). Samples were weighted and progressively heated at 105 °C, 550 °C and 980 °C, to estimate water, total organic matter including sulphides (TOM + s), the carbonate fraction (CaCO_3_ + S_2_^−X^ + SO_4_^−2X^) (including sulphides and sulphates and the residuum^100^. The residuum includes siliceous compounds, oxides, and ash deriving from complete charring of the fuel (i.e., inorganic compounds), apart from the carbonatic ash, measured as an aliquot of CaCO_3_ (Supplementary Fig. S2).

### Magnetic susceptibility

Magnetic susceptibility was measured on 130 points along the Fimon PD and 277 points along the Fimon TdA cores (Fig. 1 and Supplementary Fig. S1) with a Bartington MS2 susceptibility meter equipped with a MS2E sensor. Data were used to characterize stratigraphic units, as a proxy for clastic input and for correlating the two sequences (Fig. S1).

### Microbotanical and gradient analysis

209 samples were analysed for their microbotanical content and prepared using standard methods (including HF and acetolysis) after adding *Lycopodium* tablets for pollen and charcoal concentrations and influx estimates^101^ at the Lab. of Palynology and Paleoecology of CNR-IGAG in Milano. Pollen identification was carried out at the lowest taxonomic level possible at 400×, 630× and 1000× magnifications under a Leica DM-LB light microscope, using atlases^102–104^ and the CNR reference collection. Pollen diagrams were drawn using Tilia ver. 2.6.1^105^ and Corel Draw X8 for further graphic elaborations. The pollen sum used for % calculations includes trees, shrubs, and all upland herbs. Aquatics and wetland species are excluded. A mean pollen count of 454 ± 248 grains has been reached. Pollen zonation was obtained through constrained incremental sum of squares cluster analysis (Cavalli Sforza's chord distance as dissimilarity coefficient—CONISS^106^. Clustering, restricted to taxa whose pollen reached over 2%, is represented by the dendrogram in Fig. 2. A principal component analysis (PCA) was performed on the covariance matrix of log-transformed % selected data (terrestrial pollen taxa > 2%) (Fig. 3). Data standardization and ordination were carried out with the Vegan package^107^ in R environment^108^. Calculation of correlation coefficients and *p-*values on the same dataset was done in R using the function rcorr (package “Hmisc”)^109^ and the function corrplot (package “corrplot”)^110^ to plot the correlation matrix (Supplementary Fig. S5–S6). Such analyses allow us to extract the main eco-gradients and to detect correlations between terrestrial taxa in order to refine their ecological grouping, as shown in Figs. 6 and 7.

### Modern analogue technique and pollen indicator taxa

The Modern Analogue Technique (MAT) is a widely used statistical approach based on matching individual fossil pollen spectra to a large number of modern pollen assemblages using similarity measures^111^. Given that modern pollen analogues share similar combinations of taxa originating from comparable plant assemblages, the vegetation parameters of a modern pollen sample can be applied to any fossil sample with similar palynological composition. We used MAT and squared chord distance (SCD) to calculate dissimilarity between pairs of pollen spectra. For eco-regions prediction, only one ‘best analogue’ with the lowest SCD value was selected^112^. Aquatic and wetlands plants, spores and algae were excluded from the MAT analysis. A selection of the entire EMPD2 dataset (Eurasian Modern Pollen Database vers. 2)^39^ consisting of 5925 surface samples and 53 modern pollen assemblages from the Southern Urals collected in 2007 were selected and used as a modern calibration set, covering a long climate gradient across Eurasia. We excluded samples located in the Anatolian and Mediterranean biogeographical regions defined according to EEA (https://www.eea.europa.eu/data-and-maps/data/biogeographical-regions-europe-3). Most of the herbaceous taxa included in this Eurasian calibration set are harmonized at family or subfamily level (e.g., Asteroideae, Apiaceae, Rubiaceae). This high harmonization level together with an uneven spatial sampling distribution of the modern samples still represents a limitation in the use of MAT and may cause non-analogue conditions. For this reason, climate-sensitive pollen indicators were also used to define past floristic structures and climatic requirements especially for herbaceous-structured vegetations (*e.g*., *Xeranthemum inapertum* type, *Galium* type).

Analogue computations were performed using the R package ‘rioja’^113^. The optimal maximum number of analogues, k = 4, was determined by leave-one-out (LOO) cross-validation.

### Vegetation data

Modern pollen analogues were assigned to ecoregions based on their geographical coordinates and elevation using the Ecoregions2017 Resolve map^43^. These analogues were grouped into four time-periods corresponding to pollen zonation: 60–42 ka (pollen zone FPD1), 42–39 ka (FPD2), 39–31 ka (FPD3) and 31–27 ka (FPD 4), and the proportions of ecoregions with the best modern analogues for each pollen zone were calculated (Fig. 4). For the Altai-Sayan region in southern Siberia, where a large proportion of modern analogues was identified, plant species records from plots established at each site where surface pollen was sampled^15,41,114^ were used to interpret the vegetation and landscape of the Fimon PD fossil samples. For surface pollen samples without detailed vegetation descriptions we integrated information using “sample context” and “vegetation description” notes available in the EMPD2 dataset. These data were used to interpret vegetation types and landscape structure throughout MIS 3 at the Fimon site.

### Sieved charcoal particles analysis

Three different size ranges (62–125 µm, 125–500 µm and > 500 µm) of sieved charcoal particles were separated in 415 sediment samples of approximately 2 cm^3^ at contiguous 0.5–2.5 cm intervals using standard sieving methods^115^. Samples were gently disaggregated in a 50/50 solution of 10% sodium hexametaphosphate (NaPO_3_)_6_, and 12.5% sodium hypochlorite (NaClO) for 24 h and sieved (62, 125, 500-μm mesh). The sieved fractions were counted on a gridded platform using a stereomicroscope. Macroscopic charcoal particles (> 125 μm) are assumed to record high severity fires within a few kilometers from the study site^115,116^. Finer charcoal particles (62–125 µm size) are more widely dispersed and may represent a reliable proxy of regional fire variability (at least within 150 km) as supported by dispersal models^117^. We obtained series of charcoal concentrations (particles cm^−3^) converted into total charcoal accumulation rates (CHAR, particles cm^−2^ year^−1^) by multiplying these values by sediment accumulation rates (cm year^−1^) inferred from the age–depth model (Supplementary Fig. S7). The CHAR record (particles > 125 µm) was then decomposed into background (C_back_) and peak component using the method implemented in CharAnalysis 0.9 software^118^. Peaks, which are positive deviations from the C_background_ represent input of charcoal as a result of large, high-severity fires relatively close (1–3 km radii) to coring location, whereas total CHAR correlates best with area burned at distances from 10^0^–10^1^ km, up to an order of magnitude greater than distances defining the optimal spatial scale for peak-inferred fires^116^. The C_background_ component was determined using a moving mode robust to outliers with a 500 years window width. A Gaussian mixture model was used to identify threshold values for peak identification (0.95 percentile). The fire frequency (FF) is the total number of fires within a 1000-year window. Fire return interval (FRI) is the time between two adjacent fire events. A Signal to Noise Index (SNI) was used to evaluate the suitability of sediment-charcoal records for reconstructing local fires. The SNI compares the variability in the signal population, var (S), to the variability in the noise population, var (N): SNI = var(S)/var(S) + var(N). A SNI greater than 3 consistently identifies records appropriate for peak detection^44^.

## Supplementary Information

Supplementary Information.

## Data Availability

All data generated or analysed during this study are included in this published article and its Supplementary Information files.
