# Supplementary material for: High-resolution ecosystem changes pacing the millennial climate variability at the Middle to Upper Palaeolithic transition in NE-Italy
Source: Sci Rep. 2023 Aug 1;13:12478. doi: 10.1038/s41598-023-38081-1 (PMC10394073; doi:10.1038/s41598-023-38081-1)
Supplement: Supplementary file 1 — Supplementary Information. [file 41598_2023_38081_MOESM1_ESM.pdf]

Supplementary information

High-resolution ecosystem changes pacing the millennial climate variability at the Middle to Upper Palaeolithic transition in NE-Italy

Federica Badino[1,2*], Roberta Pini[2], Cesare Ravazzi [2], Milan Chytrý[3], Paolo Bertuletti[2], Eugenio Bortolini[1], Lydie Dudová[4], Marco Peresani[5,2], Matteo Romandini[1], Stefano Benazzi[1]

[1] Department of Cultural Heritage, University of Bologna, 48121 Ravenna, Italy

[2] CNR - Institute of Environmental Geology and Geoengineering, Lab. of Palynology and Palaeoecology, Research Group on Vegetation, Climate and Human Stratigraphy, 20126 Milano, Italy

[3] Department of Botany and Zoology, Faculty of Science, Masaryk University, Brno, Czech Republic

[4] Department of Paleoecology, Institute of Botany, Czech Academy of Sciences, Brno, Czech Republic

[5] Department of Humanities, Prehistoric and Anthropology Sciences, University of Ferrara, 44100, Ferrara, Italy

* Corresponding author: Federica Badino federica.badino@igag.cnr.it

University of Bologna,

Department of Cultural Heritage

48121 Ravenna, Italy

http://www.erc-success.eu/

SI-1

Fimon basin: lithostratigraphical and local environmental setting

Lake Fimon (22 m a.s.l.) is located in a wide basin in the northern sector of the Berici Hills, a karstic plateau 200 km^2 wide, emerging from the alluvial plain (**Fig. 1**)[1]. The Berici Hills belong to the so called Euganeo-Berico-Lessinian wedge[2], a structural high slightly affected by the Neoalpine compression during the Miocene–Quaternary leading to the exhumation of the Berici Hills and bounded to the east by the NW–SE Schio-Vicenza fault[3]. The plateau is made of Eocene marl and Oligocene limestone, cut by Oligocene mafic volcanic dykes[4]. Younger deposits and residual soils are confined to the karstic sinkholes[5].

Three cores were drilled through the sedimentary fill of the Fimon Valleys at different locations along a transect from the external sector (open towards the alluvial plain) to the innermost site occupied by Lake Fimon (**Fig. 2c**):

1. Torri di Arcugnano core (Fimon TdA: 48,60 m deep), external sector of the Fimon Valleys (**Fig. 1c and S1**);
2. Ponte Debba core (Fimon PD: 44.50 m deep) close to the northern lake shore (**Fig. 1c and S1**);
3. Fimon Lake core (Fimon FL: 16.36 m deep) drilled from the lake surface (**Fig. 1c**).

Fimon PD and Fimon TdA cores were sampled for pollen analysis, AMS datings, geochemical analysis (LOI), sand petrography, magnetic susceptibility[6–8] and finely correlated (**Fig. S1**).

The sedimentological structures observed in Fimon PD (21.75 - 19.39 m) and TdA (31 - 26 m) core sections and the high-resolution record of limnic algae and aquatic plants from Fimon PD core (**Fig. S2**) suggest that deposition took place in a shallow lacustrine environment for most of the MIS 3 interval (FL3 lithostratigraphic zone, **Fig. S1-2**). In this context, massive grey clay intervals containing very high siliceous + oxides values (90% of the dry weights) alternate with several organic-rich (silty gyttja) layers marked by flooded littoral zones expansion suggesting a deepening of the water column (FL3b, e, h, g and i; **Fig. S2**). A major variation of the water column height is marked by aquatic algae (p.m.p. *Pediastrum* and *Botryococcus*) and carbonate fraction increase (20.08-20.30 m, FL 3h-FL4; **Fig. S2**). The interval between 19.90-19.39 m is made of peat deposits mainly formed by thin layers of Cyperaceae leaves and characterized by high total organic matter content including sulphides (30-50%; FL5 in **Fig. S2**). The peat interval is attributed to long-lasting

marshy conditions occupying a wide area as recorded by the finding of the same peat layer in the three studied cores[8,9]. During this phase accumulation rates increased from ca. 0.006 to 0.02 cm/years due to favourable (local) biomass growth under relatively humid conditions[10]. A sequence of laminated minerogenic silts rich in limnic algae (FL6; **Fig. S2**) overlayed these terrestrial organic deposits indicate a freshwater input that drowned the mire (see [10] for further information).

SI-2

Archaeological sites context

Fumane

Grotta di Fumane is located at the foot of the Venetian Prealps in the western Lessini Mountains at an altitude of 350 m and forms a part of a fossil karst system composed of several cavities in dolomitic limestone. This karst complex preserves a well-documented sedimentary sequence 12 m in thickness, divided into four main macro-units based on the lithological features and archaeological remains. From the bottom to top, these four macro-units are distinguished as a sand unit (S), a breccia unit (BR), an anthropogenic unit (A) and a landslide unit (D)[11]. Macro-unit A, which is the focus of our study, includes several horizontal layers from A13 to A1, ranging from layers dominated by residual dolomite sands (layers A13–A12), angular fine to medium-sized stone layers (levels in the A10 complex, layers A7, A4), stones, slabs and fine material (layer A3) or anthropogenic material (A11, levels in A10 and A9 complexes, A6, A2–A1)[12]. Unit A shows evidence of much more intense human occupations, which include (Final) Mousterian (A11, A10 A9, A6–A5, A4), Uluzzian (A3) and Proto-Aurignacian (A2–A1) assemblages[13–15]. Landslide Unit D mostly consists of large blocks, which collapsed from the cavity's roof because of macrogelivation processes. Evidence of human occupation is present within the lowermost layers D3d, D3b and D3a, representing the latest Aurignacian units [16] and becoming sporadic in the middle-level D1d, where some Gravettian artefacts have been detected [17]. Research at Fumane is coordinated by University of Ferrara (M.P.) in the framework of a project supported by the Ministry of Culture – “SABAP per le province di Verona, Rovigo e Vicenza”, public institutions (Lessinia Mountain Community - Regional Natural Park, Fumane Municipality, BIMAdige, SERIT) and by private institutions, associations and companies. Research campaigns 2017 and 2019 have received funding from the

European Research Council (ERC) under the European Union's Horizon 2020 research and innovation programme (grant agreement No 724046 – SUCCESS, http://www.erc-success.eu).

Broion shelter

Riparo del Broion is situated at 135 m a.s.l. at the base of a steep cliff of Mount Brosimo (327 m a.s.l.) along a terraced slope (Berici Hills). The shelter is 10 m long, 6 m deep and 17 m high and originated from rock collapse along a major ENE-WSW oriented fault that developed from thermoclastic processes and chemical dissolution comparably to other cavities in the area [18]. Two additional Paleolithic cavities were investigated on the western side of the same cliff, Grotta del Buso Doppio del Broion and Grotta del Broion[19]. Archaeological excavations were initially directed by Alberto Broglio (1998 -2008) and by two of us (M.P. and M.R.) in 2015 on a 20sqm area bounded to north and west from the rock walls. Faunal remains and Middle and Upper Paleolithic (Uluzzian, Gravettian and Epigravettian) cultural material was uncovered [20–22]. The bedrock has not yet been reached. Sediments are mostly small stones and gravel with large prevalence on loams: 16 stratigraphic units planarly bedded have been identified. The lowermost (11, 9, 7 and 4) contain Mousterian artefacts, faunal remains and clearly differentiate in dark-brownish color from the other units.

Research at Riparo Broion is coordinated by the Bologna (M.R.) and Ferrara (M.P.) Universities in the framework of a project supported by the Ministry of Culture – "SABAP per le province di Verona, Rovigo e Vicenza", public institutions (Longare Municipality), institutions (Leakey Foundation, Spring 2015 Grant; Istituto Italiano di Preistoria e Protostoria). Research campaigns 2017-2019 have received funding from the European Research Council (ERC) under the European Union's Horizon 2020 research and innovation programme (grant agreement No 724046 – SUCCESS, http://www.erc-success.eu).

San Bernardino

Grotta Maggiore di San Bernardino (Mossano, Berici Hills, Vicenza), located at 135 m a.s.l., lies in the foreland of the eastern Italian Alps. The cave opens along the eastern slope of the karst plateau of the Berici Hills on a carbonate sandstone cliff from the Middle Eocene, facing the alluvial plain of the Bacchiglione River and the south-western sector of the Euganean Hills. The cave is 41 m long, 7 m wide and 9 m high and was produced by

thermoclastic processes and chemical dissolution, which widened deep, SE-NW oriented fractures. In the western side of the same slope, a second, shorter cave named Grotta Minore di San Bernardino is situated. The first archaeological excavations were carried out by Prof. P. Leonardi in the 1960s in the area facing the medieval wall, uncovering a Pleistocene sequence with faunal remains and knapped stones[23,24]. A second cycle of research, between 1986 and 1995, allowed a more detailed reexamination of the different facies of the stratigraphic series between the inner and outer zones of the cave[25]. At present, the sedimentary sequence is 4.5 m thick and includes eight stratigraphic units with sub-horizontal bedding, which tilt progressively outside the cave. Excavations from 1986 to 1994 confirmed that all the units contained Mousterian industries except unit I, which contained a few Upper Palaeolithic artifacts. Neanderthals used San Bernardino Cave as a place where carcasses processing was finalized, after an initial process at the kill-site, and then prepared for consumption[26].

SI-3

Archaeozoological data

Analysis carried out on Late Mousterian levels at Fumane Cave in Lessini Prealps and San Bernardino Cave and Broion Shelter in the Berici Mounts, indicate a prevalence of cervids (e.g., *Cervus elaphus*, *Capreolus capreolus, Megaloceros giganteus*) over caprids (*Capra ibex* and *Rupicapra rupicapra*) and bisons (*Bison priscus* and *Bos primigenius*)[26–31]. The presence of freshwaters is also evidenced by findings of beaver and moose remains. Afterwards, between ca. 44-39 ka, major ecological changes occurred in faunal assemblages. In stratigraphic reference sequences we notice a consistent decrease of *Cervus elaphus* and *Capreolus capreolus* as opposed to a sharp increase in *Capra ibex* and *Rupicapra rupicapra* (see the Late Mousterian/Uluzzian/ Protoaurignacian transition, at Fumane cave) and the occurrence of cold-adapted bird species currently distributed at high latitude in the Boreal hemisphere[27,28,30]. The presence of bovids *(Bos/Bison)* is constant, while moose and giant deer are less frequent and well attested in Mousterian and Uluzzian layers of all three sites and in open sites (see Settepolesini[32]) during the *Middle Würm*. Wild boar rarer yet, is more abundant at lower elevations (Grotta di San Bernardino, Mousterian Units II + III; Riparo del Broion, Uluzzian layers 1e+1f+1g). The presence of woolly rhinoceros (*Coelodonta antiquitatis*) in the Uluzzian layer A3 at

Fumane Cave and of *Stephanorhinus* sp. at San Bernardino Cave indicate a decreasing temperature trend. This is also supported by the increase in abundance of a variety of carnivores taxa in the Uluzzian unit[28,33] and, particularly, the presence of wolverine (*Gulo gulo*), ermine (*Mustela erminea*), and arctic fox (*Alopex* cfr. *lagopus*). Mountain hare (*Lepus timidus*) also appeared between lagomorph's[28].
Between 39-31 ka (FPD3), the degrowth of cervids becomes a collapse, while the abundance of ibex and chamois reached its *acme* (see Fumane cave). In this chrono-phase, at Fumane Cave, mountain hare (*Lepus timidus*) is still present[27,28,30].
As regards the alluvial plain of the Po River, Prof. Gallini and Sala B. wrote about Settepolesini in the Middle Würm (ca. 35,000 – 33,000 years ago): *"The findings of mammoth and Irish elk, ascribed by the radiocarbon dating's to this period, together with woolly rhinoceros, steppe bison and elk."*[32].
Between 31 to 27 ka, faunal data from caves and shelters around the Berici Hills record the occurrence of *Alces alces*, fishes and water bird remains, pointing to the presence of water bodies. Marmots, ibex and chamois also occured [19,29,34–36]. As for birds, boreal species that possibly sought refuge in the Mediterranean areas during colder phases, are documented at Fumane cave[30]. The subsequent climatic deterioration linked to the LGM initiation at 27.3 ka[37] could have been responsible, together with an increasing human pressure, to the later extinction of cave bears in Northeastern Italy[38,39].

All these phases are well detailed by the analysis of bird assemblage at Fumane cave. Several identified bird species (rock ptarmigan, black grouse, Boreal owl, bearded vulture, white-backed woodpecker, red and yellow-billed chough, northern nutcracker and white-winged snow finch, currently live in Italy at considerably higher altitudes than Fumane cave. The presence of their fossil remains at 350 m asl suggests the downward shift of the vegetation belts during MIS 3. The presence of remains probably belonging to willow grouse in layer A6 and to parrot crossbill in layers A1+A2 might be an example of two boreal species seeking a *refugium* in Mediterranean Europe[35,40].

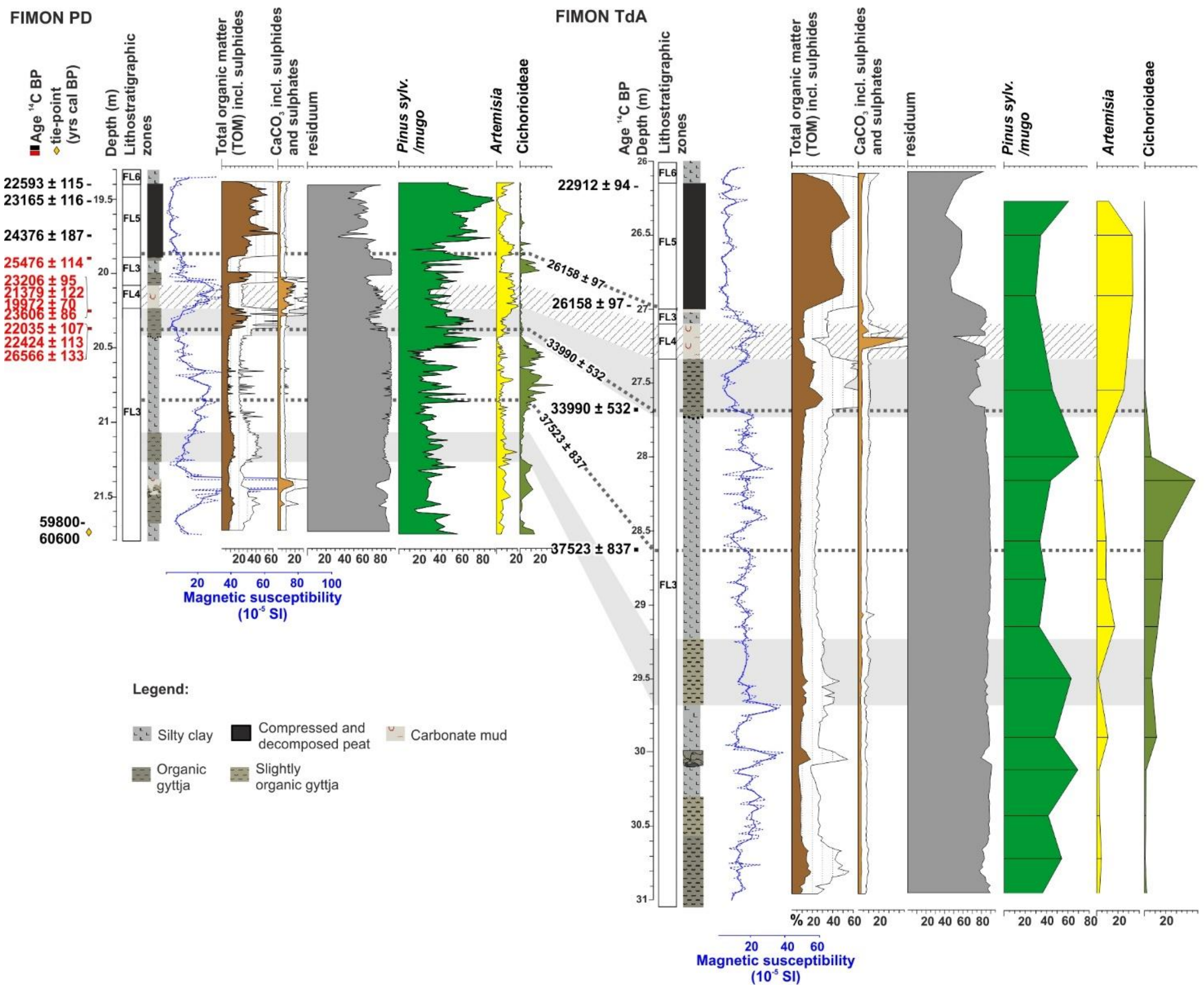

Figure S1. Litho-, chrono- and biostratigraphic correlation of Fimon PD and Fimon TdA cores, plotted versus their original depth. ^{14}C ages are plotted according to their stratigraphic position. Radiocarbon ages indicated in red were excluded from our modelling since they mostly yielded age reversals, as previously demonstrated[8,41]. Magnetic susceptibility, LOI-determined total organic matter including sulphides, $CaCO_3$ including sulphides and sulphates and residuum, accompanied by the percentage curves of *Pinus sylvestris/mugo* type, *Artemisia* and Cichorioideaea were used to correlate the sequences. Also, data shows the reproducibility of the two Lake Fimon records.

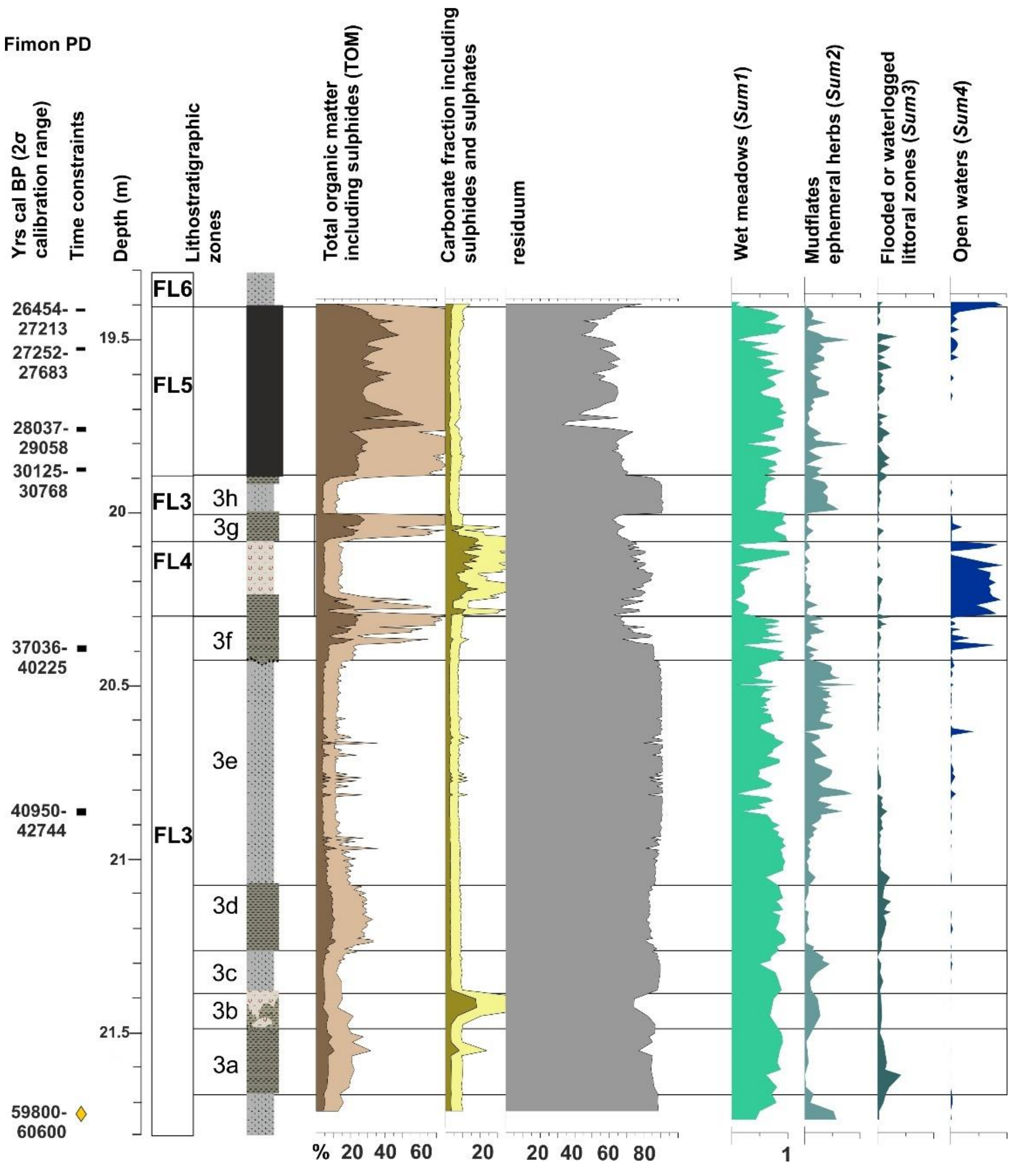

Figure S2 - Fimon PD geochemical data and local environments. Normalized Sum1: wet meadows (*Filipendula*, *Thalictrum* and *Lythrum salicaria*); Sum2: mudflates ephemeral herbs (Cichorioideae and *Xeranthemum inapertum* type); Sum3: flooded or waterlogged littoral zones (*Anagallis*, *Lythrum portula*, *Sparganium emersum* type, *Ceratophyllum*, *Typha latifolia* type); Sum4: open waters (*Nymphaea, Nuphar, Myriophyllum verticillatum, Myriophyllum spicatum, Myriophyllum alterniflorum, Spirogyra, Spirogyra scrobiculata, Zygnemataceae, Mougeotia, Pediastrum, Botryococcus, Scenedesmus, Gloeotrichia*).

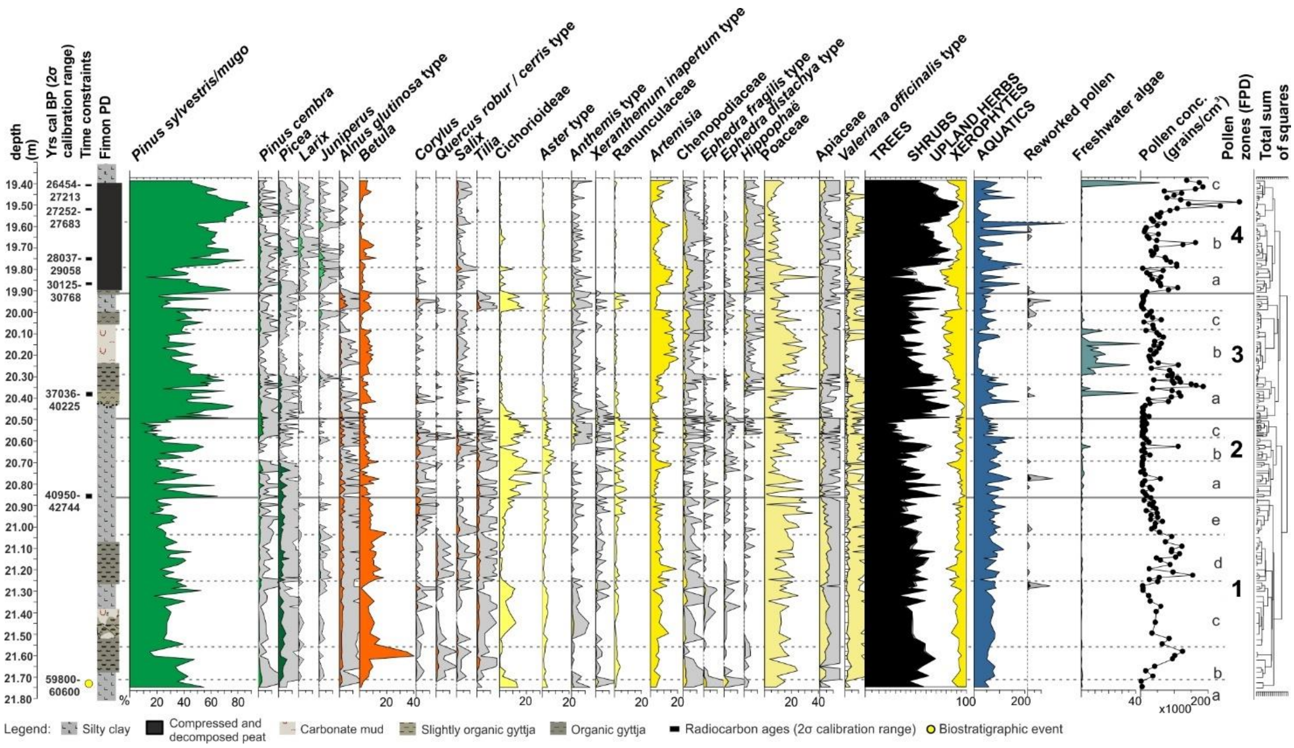

Figure S3. Selected terrestrial percentage curves from the Fimon PD pollen record. Aquatics and wetland are excluded from the pollen sum.

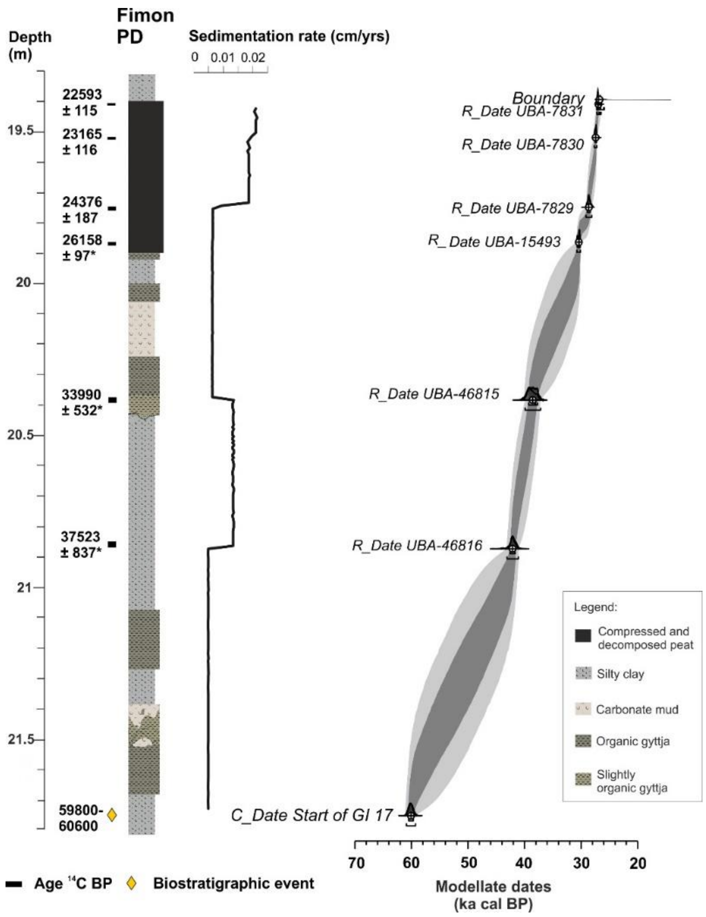

Figure S4. Fimon PD age-depth model calculated with the OxCal 4.4 calibration software[42] using IntCal20 calibration curve[43].

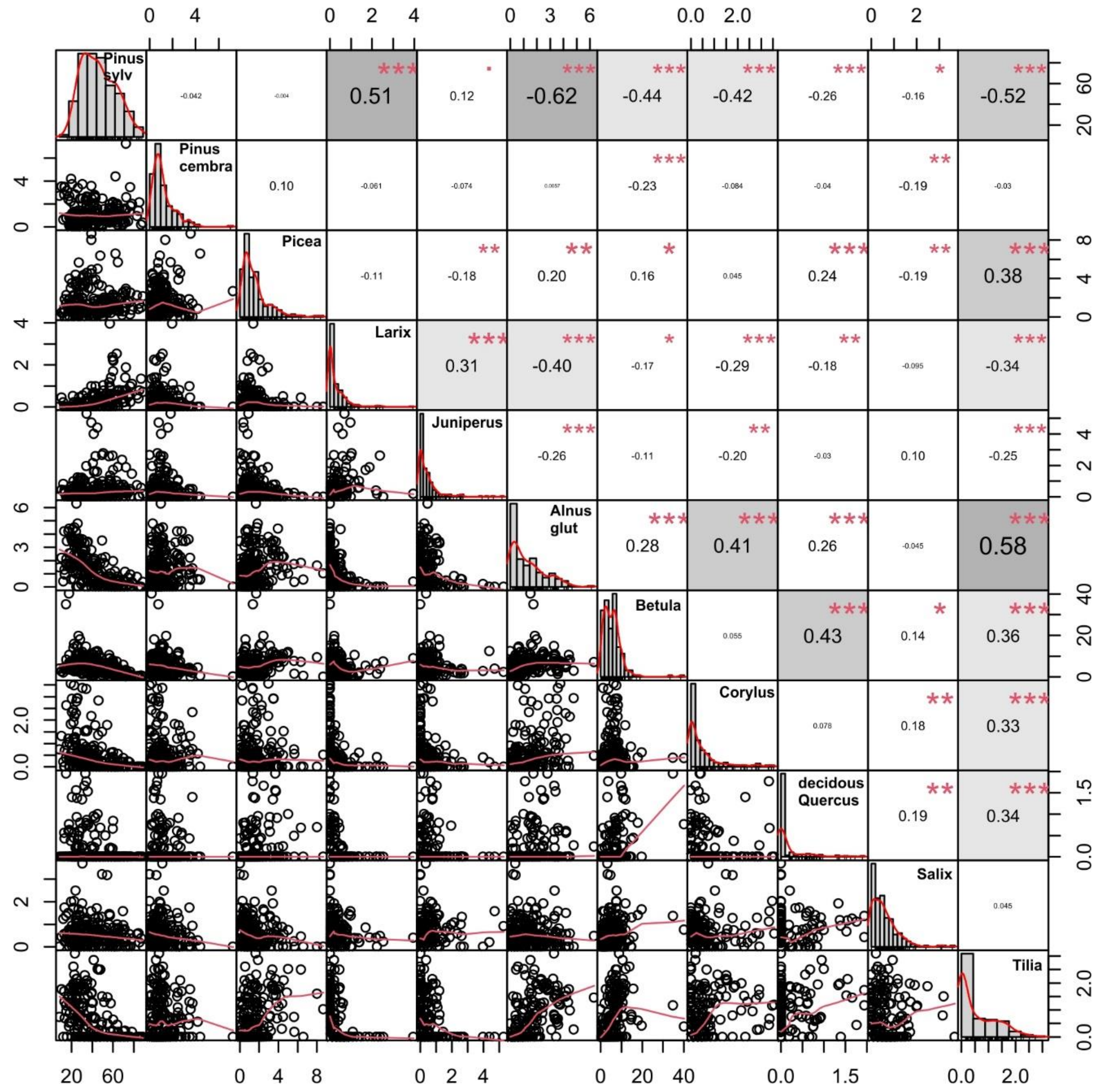

Figure S5. Correlation matrix of Fimon PD woody taxa > 2%.

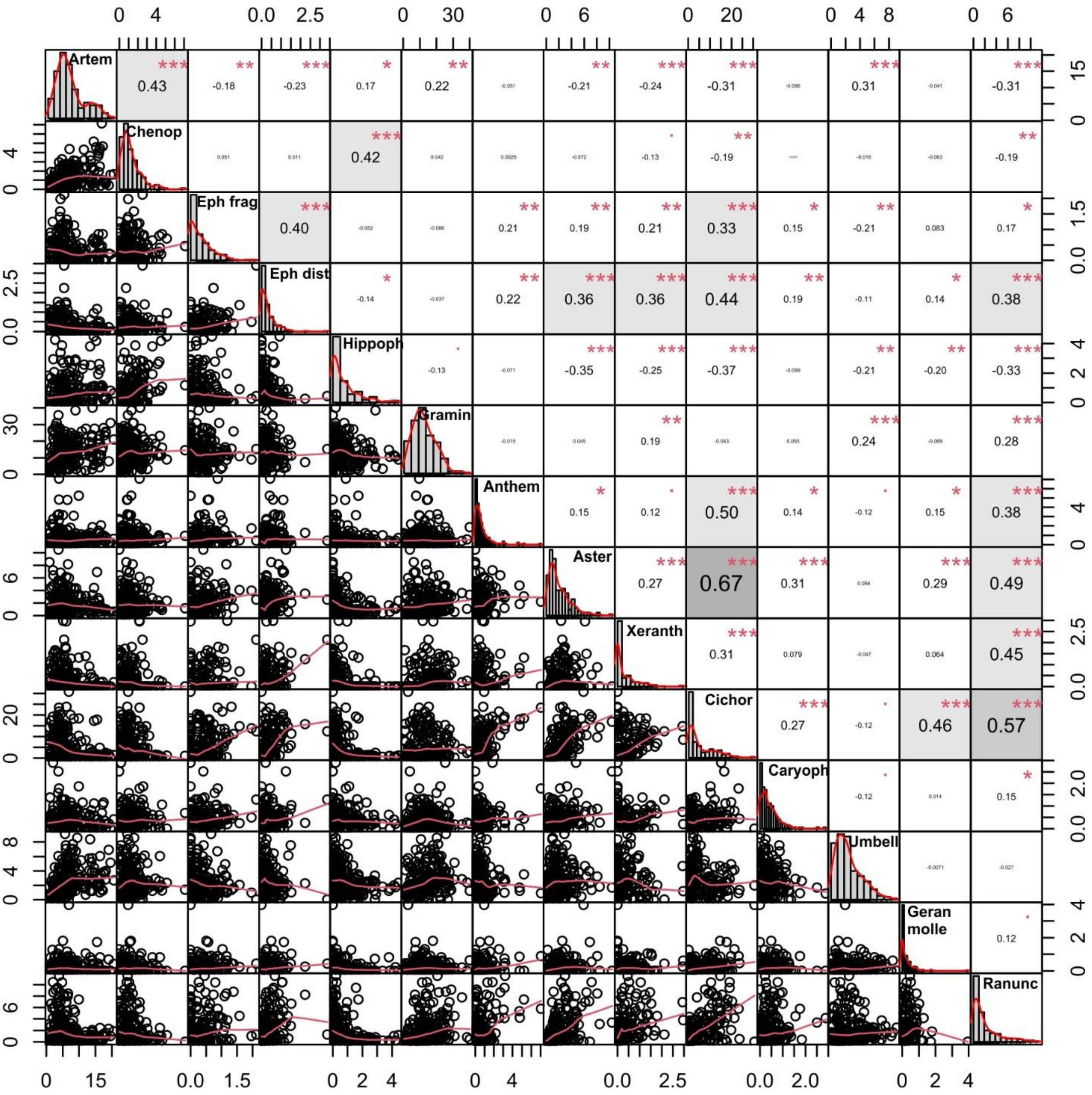

Figure S6. Correlation matrix of Fimon PD upland herbs > 2%.

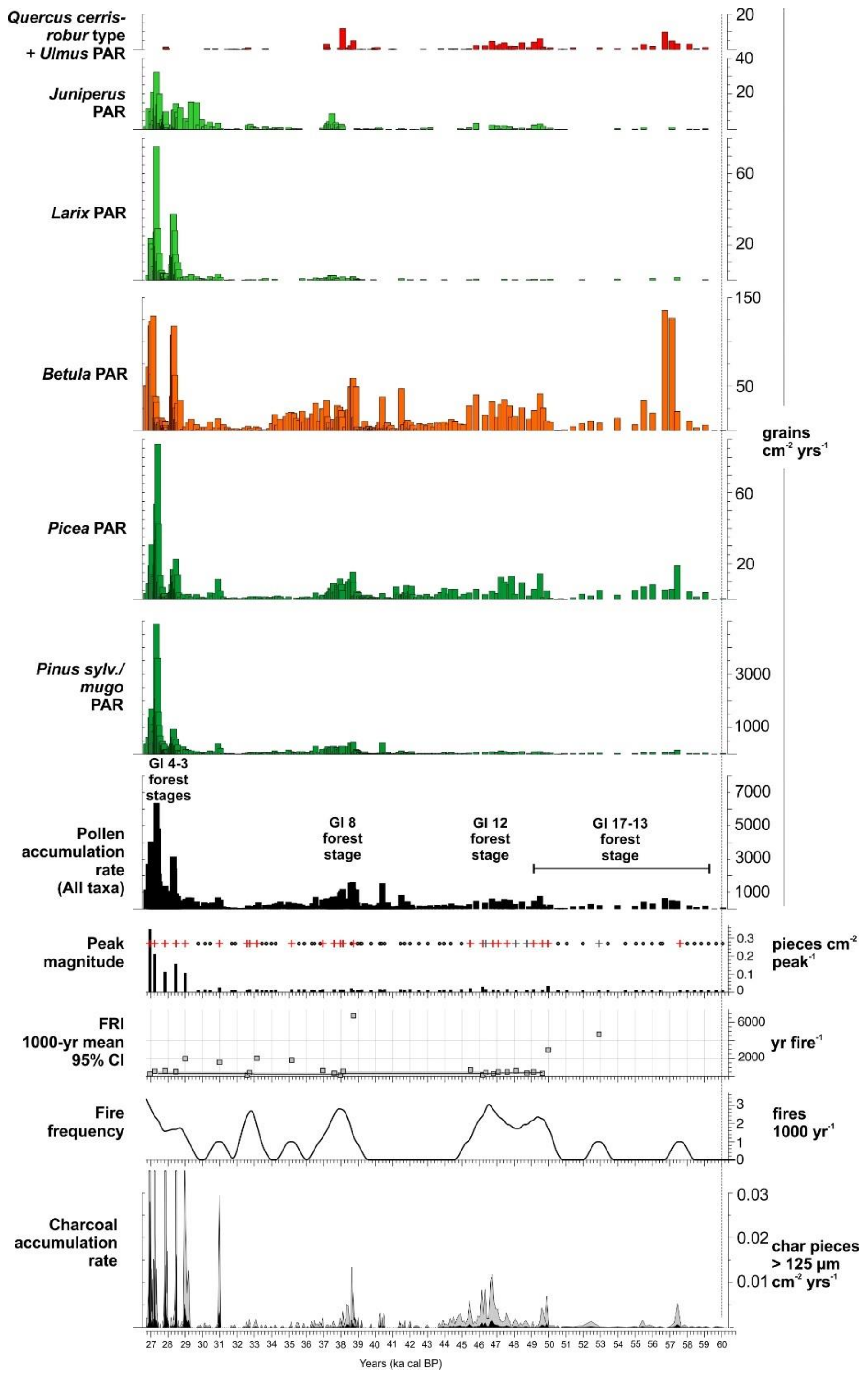

Figure S7. From bottom to the top: Macroscopic charcoal accumulation rates (charcoal pieces >125 micron cm-^{2}yr^{-1}; grey curve); Fire frequency (FF): the total number of fires within a 1000-year window; Fire return interval (FRI, 1000-yr mean) and 95% CI: the time between two adjacent fire events; Peak magnitude (pieces cm^{-2} peak^{-1}); Local fires are indicated by red crosses (positive passing the charcoal peak screening and SNI>3) while other peaks: grey crosses (positive passing the charcoal peak screening and SNI<3) and grey dots (negative passing the charcoal peak screenin), identified using the method implemented in CharAnalysis 0.9[44]. Histograms show Pollen

Accumulation Rates (PAR) of all pollen taxa and selected pollen taxa (*Pinus sylv./mugo*, *Picea*, *Betula*, *Larix*, *Juniperus*, *Quercus cerris/robur* type + *Ulmus*).

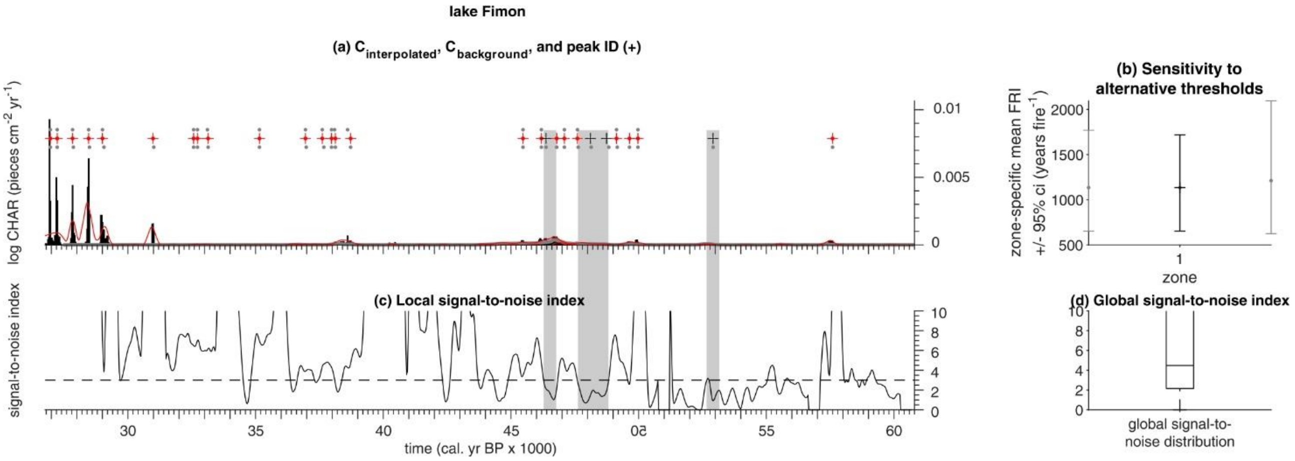

Figure S8. Boxplot of all Signal to Noise (SNI) values[45]. Analysis were carried out using the method implemented in CharAnalysis 0.9 software[44].

FIMON PD record		METADATA FROM EMPD2						ECOZONES AND BIOMES	
**Fossil samples - pollen zones**	**Fossil samples - modelled mean ages**	**First analogue - Sample Name**	**Longitude**	**Latitude**	**Elevation**	**SampleContext**	**VegDescription**	**ECOZONE_NAME**	**BIOME_NAME**
FPD4	26752	Pidek_a5	23,06306	50,58694	279	closed forest	Nemoral mixed forest, with Picea, Abies, Fagus - national park	Central European mixed forests	Temperate Broadleaf & Mixed Forests
FPD4	26871	Kunes_a59	87,64611	50,30750	1342	treeless vegetation	Saline grassland	Altai alpine meadow and tundra	Montane Grasslands & Shrublands
FPD4	27001	Kunes_a46	87,80139	50,10583	2066	treeless vegetation	Mesic grassland	Altai alpine meadow and tundra	Montane Grasslands & Shrublands
FPD4	27047	Kunes_a87	87,89028	50,30417	2319	treeless vegetation	Tundra/Dryas	Altai alpine meadow and tundra	Montane Grasslands & Shrublands
FPD4	27094	Kunes_a97	85,67556	50,98500	1382	closed forest	Taiga/Pinus sibirica-Picea	Altai montane forest and forest steppe	Temperate Conifer Forests
FPD4	27140	Kunes_a45	87,78972	50,09417	2094	closed forest	Hemiboreal forest/Pinus sibirica	Altai alpine meadow and tundra	Montane Grasslands & Shrublands
FPD4	27185	Ortu_b49	6,98400	44,68300	2570	0	0	Alps conifer and mixed forests	Temperate Conifer Forests
FPD4	27231	Kunes_a43	87,80472	50,11472	2013	closed forest	Hemiboreal forest/Pinus sibirica-Larix	Altai alpine meadow and tundra	Montane Grasslands & Shrublands

FPD4	27277	Kunes_a43	87,80472	50,11472	2013	closed forest	Hemiboreal forest/Pinus sibirica-Larix	Altai alpine meadow and tundra	Montane Grasslands & Shrublands
FPD4	27323	Binney2017_E2846	104,32000	51,47000	475	0	0	East Siberian taiga	Boreal Forests/Taiga
FPD4	27369	Binney2017_E2846	104,32000	51,47000	475	0	0	East Siberian taiga	Boreal Forests/Taiga
FPD4	27415	Binney2017_E2846	104,32000	51,47000	475	0	0	East Siberian taiga	Boreal Forests/Taiga
FPD4	27464	Binney2017_E2846	104,32000	51,47000	475	0	0	East Siberian taiga	Boreal Forests/Taiga
FPD4	27515	Ortu_b49	6,98400	44,68300	2570	0	0	Alps conifer and mixed forests	Temperate Conifer Forests
FPD4	27568	Ryabogina_a27	65,54812	56,48371	64	wetland	near-water vegetation (reeds, sedges, duckweed)	Western Siberian hemiboreal forests	Temperate Broadleaf & Mixed Forests
FPD4	27620	Binney2017_E2863	107,40000	53,20000	480	0	0	East Siberian taiga	Boreal Forests/Taiga
FPD4	27671	Ortu_b49	6,98400	44,68300	2570	0	0	Alps conifer and mixed forests	Temperate Conifer Forests
FPD4	27724	Binney2017_E2728	104,72000	52,08000	475	0	0	East Siberian taiga	Boreal Forests/Taiga
FPD4	27776	Binney2017_E3546	129,33000	62,00000	200	0	0	East Siberian taiga	Boreal Forests/Taiga
FPD4	27828	Kunes_a87	87,89028	50,30417	2319	treeless vegetation	Tundra/Dryas	Altai alpine meadow and tundra	Montane Grasslands & Shrublands
FPD4	27880	Kunes_a46	87,80139	50,10583	2066	treeless vegetation	Mesic grassland	Altai alpine meadow and tundra	Montane Grasslands & Shrublands
FPD4	27932	Kunes_a49	87,80000	50,10583	2085	treeless vegetation	Meadow steppe	Altai alpine meadow and tundra	Montane Grasslands & Shrublands

FPD4	27984	Kunes_a87	87,89028	50,30417	2319	treeless vegetation	Tundra/Dryas	Altai alpine meadow and tundra	Montane Grasslands & Shrublands
FPD4	28036	Binney2017_E3546	129,33000	62,00000	200	0	0	East Siberian taiga	Boreal Forests/Taiga
FPD4	28088	Kunes_a87	87,89028	50,30417	2319	treeless vegetation	Tundra/Dryas	Altai alpine meadow and tundra	Montane Grasslands & Shrublands
FPD4	28139	Kunes_a49	87,80000	50,10583	2085	treeless vegetation	Meadow steppe	Altai alpine meadow and tundra	Montane Grasslands & Shrublands
FPD4	28191	Kunes_a49	87,80000	50,10583	2085	treeless vegetation	Meadow steppe	Altai alpine meadow and tundra	Montane Grasslands & Shrublands
FPD4	28243	Kunes_a49	87,80000	50,10583	2085	treeless vegetation	Meadow steppe	Altai alpine meadow and tundra	Montane Grasslands & Shrublands
FPD4	28295	Kunes_a43	87,80472	50,11472	2013	closed forest	Hemiboreal forest/Pinus sibirica-Larix	Altai alpine meadow and tundra	Montane Grasslands & Shrublands
FPD4	28347	Kunes_a97	85,67556	50,98500	1382	closed forest	Taiga/Pinus sibirica-Picea	Altai montane forest and forest steppe	Temperate Conifer Forests
FPD4	28399	Kunes_a87	87,89028	50,30417	2319	treeless vegetation	Tundra/Dryas	Altai alpine meadow and tundra	Montane Grasslands & Shrublands
FPD4	28451	EPDcoretop_E320	60,75000	56,76667	230	wetland bog	Pinus sylvestris, Betula pubescens	West Siberian taiga	Boreal Forests/Taiga
FPD4	28503	Binney2017_E2807	106,15000	52,52000	449	0	0		
FPD4	28555	EPDcoretop_E1193	11,67833	46,76083	870	wetland bog	Typha, Phragmites, Utricularia	Alps conifer and mixed forests	Temperate Conifer Forests

FPD4	28607	Kunes_a49	87,80000	50,10583	2085	treeless vegetation	Meadow steppe	Altai alpine meadow and tundra	Montane Grasslands & Shrublands
FPD4	28710	Kunes_a87	87,89028	50,30417	2319	treeless vegetation	Tundra/Dryas	Altai alpine meadow and tundra	Montane Grasslands & Shrublands
FPD4	28863	Binney2017_E2863	107,40000	53,20000	480	0	0	East Siberian taiga	Boreal Forests/Taiga
FPD4	29017	Kunes_a43	87,80472	50,11472	2013	closed forest	Hemiboreal forest/Pinus sibirica-Larix	Altai alpine meadow and tundra	Montane Grasslands & Shrublands
FPD4	29172	Kunes_a43	87,80472	50,11472	2013	closed forest	Hemiboreal forest/Pinus sibirica-Larix	Altai alpine meadow and tundra	Montane Grasslands & Shrublands
FPD4	29326	Kunes_a59	87,64611	50,30750	1342	treeless vegetation	Saline grassland	Altai alpine meadow and tundra	Montane Grasslands & Shrublands
FPD4	29481	Kunes_a59	87,64611	50,30750	1342	treeless vegetation	Saline grassland	Altai alpine meadow and tundra	Montane Grasslands & Shrublands
FPD4	29635	Kunes_a20	87,89639	50,17472	1534	treeless vegetation	Dry steppe	Altai alpine meadow and tundra	Montane Grasslands & Shrublands
**FPD4**	**29789**	**Kunes_a87**	**87,89028**	**50,30417**	**2319**	**treeless vegetation**	**Tundra/Dryas**	**Altai alpine meadow and tundra**	**Montane Grasslands & Shrublands**
FPD4	29943	Kunes_a87	87,89028	50,30417	2319	treeless vegetation	Tundra/Dryas	Altai alpine meadow and tundra	Montane Grasslands & Shrublands
FPD4	30096	Kunes_a151	88,01139	49,94556	2570	treeless vegetation	Dry steppe	Altai alpine meadow and tundra	Montane Grasslands & Shrublands

FPD4	30250	Kunes_a87	87,89028	50,30417	2319	treeless vegetation	Tundra/Dryas	Altai alpine meadow and tundra	Montane Grasslands & Shrublands
**FPD4**	**30405**	**Kunes_a87**	**87,89028**	**50,30417**	**2319**	**treeless vegetation**	**Tundra/Dryas**	**Altai alpine meadow and tundra**	**Montane Grasslands & Shrublands**
**FPD4**	**30561**	**Kunes_a49**	**87,80000**	**50,10583**	**2085**	**treeless vegetation**	**Meadow steppe**	**Altai alpine meadow and tundra**	**Montane Grasslands & Shrublands**
FPD4	30717	Kunes_a87	87,89028	50,30417	2319	treeless vegetation	Tundra/Dryas	Altai alpine meadow and tundra	Montane Grasslands & Shrublands
FPD4	30875	Kunes_a43	87,80472	50,11472	2013	closed forest	Hemiboreal forest/Pinus sibirica-Larix	Altai alpine meadow and tundra	Montane Grasslands & Shrublands
FPD4	31032	Tonkov_o1	23,40618	41,74560	2230	treeless vegetation	Groups of Pinus mugo within patches of herb vegetation	Rodope montane mixed forests	Temperate Broadleaf & Mixed Forests
FPD3	31188	Lapteva_b5	60,84237	56,25898	295	closed forest	coniferous subtaiga. LocalVeg: pine forest	West Siberian taiga	Boreal Forests/Taiga
**FPD3**	**31425**	**Dambach_a369**	**7,93520**	**49,66130**	**415**	**0**	**0**	**Western European broadleaf forests**	**Temperate Broadleaf & Mixed Forests**
**FPD3**	**31504**	**Novenko_c7**	**38,59060**	**53,67060**	**155**	**wetland bog**	**eutrophic peatland on floodplain**	**East European forest steppe**	**Temperate Broadleaf & Mixed Forests**
FPD3	31661	Lapteva_b86	59,03333	54,10000	477	natural grassland	steppe. LocalVeg: herb-bunchgrass steppe	Kazakh forest steppe	Temperate Grasslands, Savannas & Shrublands

**FPD3**	**31819**	**Karpinska_c20**	**21,55670**	**49,99640**	**227**	**pasture**	**Cirsietum rivularis**	**Carpathian montane forests**	**Temperate Conifer Forests**
**FPD3**	**31977**	**Lapteva_a60**	**57,88907**	**54,89668**	**451**	**0**	**forest-steppe**	**Urals montane forest and taiga**	**Boreal Forests/Taiga**
**FPD3**	**32132**	**Novenko_b4**	**38,60961**	**53,67365**	**176**	**pasture**	**Northern forest steppe**	**East European forest steppe**	**Temperate Broadleaf & Mixed Forests**
**FPD3**	**32288**	**Dambach_a369**	**7,93520**	**49,66130**	**415**	**0**	**0**	**Western European broadleaf forests**	**Temperate Broadleaf & Mixed Forests**
FPD3	32445	Novenko_b6	37,62833	54,04417	242	closed forest	broad-leaved forest	East European forest steppe	Temperate Broadleaf & Mixed Forests
FPD3	32601	Kunes_a59	87,64611	50,30750	1342	treeless vegetation	Saline grassland	Altai alpine meadow and tundra	Montane Grasslands & Shrublands
FPD3	32758	Kunes_a54	91,86944	51,71028	1221	treeless vegetation	Dry steppe	Sayan Intermontane steppe	Temperate Grasslands, Savannas & Shrublands
**FPD3**	**32916**	**Ortu_d39**	**6,55700**	**44,61300**	**1067**	**closed forest**	**0**	**Northeast Spain and Southern France Mediterranean forests**	**Mediterranean Forests, Woodlands & Scrub**
FPD3	33073	Kunes_a87	87,89028	50,30417	2319	treeless vegetation	Tundra/Dryas	Altai alpine meadow and tundra	Montane Grasslands & Shrublands
FPD3	33230	Kunes_a59	87,64611	50,30750	1342	treeless vegetation	Saline grassland	Altai alpine meadow and tundra	Montane Grasslands & Shrublands
FPD3	33387	Kunes_a49	87,80000	50,10583	2085	treeless vegetation	Meadow steppe	Altai alpine meadow and tundra	Montane Grasslands & Shrublands

FPD3	33623	Kunes_a87	87,89028	50,30417	2319	treeless vegetation	Tundra/Dryas	Altai alpine meadow and tundra	Montane Grasslands & Shrublands
FPD3	33857	Kunes_a54	91,86944	51,71028	1221	treeless vegetation	Dry steppe	Sayan Intermontane steppe	Temperate Grasslands, Savannas & Shrublands
FPD3	34014	Pidek_a5	23,06306	50,58694	279	closed forest	Nemoral mixed forest, with Picea, Abies, Fagus - national park	Central European mixed forests	Temperate Broadleaf & Mixed Forests
FPD3	34170	Kunes_a59	87,64611	50,30750	1342	treeless vegetation	Saline grassland	Altai alpine meadow and tundra	Montane Grasslands & Shrublands
FPD3	34327	Kunes_a82	87,89028	50,29556	2216	treeless vegetation	Alpine grassland	Altai alpine meadow and tundra	Montane Grasslands & Shrublands
FPD3	34484	Pidek_a5	23,06306	50,58694	279	closed forest	Nemoral mixed forest, with Picea, Abies, Fagus - national park	Central European mixed forests	Temperate Broadleaf & Mixed Forests
FPD3	34798	Pidek_a5	23,06306	50,58694	279	closed forest	Nemoral mixed forest, with Picea, Abies, Fagus - national park	Central European mixed forests	Temperate Broadleaf & Mixed Forests
FPD3	34955	Kunes_a87	87,89028	50,30417	2319	treeless vegetation	Tundra/Dryas	Altai alpine meadow and tundra	Montane Grasslands & Shrublands
FPD3	35111	Kunes_a59	87,64611	50,30750	1342	treeless vegetation	Saline grassland	Altai alpine meadow and tundra	Montane Grasslands & Shrublands

FPD3	35266	Pidek_a5	23,06306	50,58694	279	closed forest	Nemoral mixed forest, with Picea, Abies, Fagus - national park	Central European mixed forests	Temperate Broadleaf & Mixed Forests
FPD3	35425	Kunes_a59	87,64611	50,30750	1342	treeless vegetation	Saline grassland	Altai alpine meadow and tundra	Montane Grasslands & Shrublands
FPD3	35583	Pidek_a5	23,06306	50,58694	279	closed forest	Nemoral mixed forest, with Picea, Abies, Fagus - national park	Central European mixed forests	Temperate Broadleaf & Mixed Forests
FPD3	35740	Kunes_a97	85,67556	50,98500	1382	closed forest	Taiga/Pinus sibirica-Picea	Altai montane forest and forest steppe	Temperate Conifer Forests
FPD3	35897	Pidek_a5	23,06306	50,58694	279	closed forest	Nemoral mixed forest, with Picea, Abies, Fagus - national park	Central European mixed forests	Temperate Broadleaf & Mixed Forests
FPD3	36053	Pidek_a5	23,06306	50,58694	279	closed forest	Nemoral mixed forest, with Picea, Abies, Fagus - national park	Central European mixed forests	Temperate Broadleaf & Mixed Forests
FPD3	36209	Pidek_a5	23,06306	50,58694	279	closed forest	Nemoral mixed forest, with Picea, Abies, Fagus - national park	Central European mixed forests	Temperate Broadleaf & Mixed Forests
FPD3	36366	Kunes_a59	87,64611	50,30750	1342	treeless vegetation	Saline grassland	Altai alpine meadow and tundra	Montane Grasslands & Shrublands

FPD3	36524	Kunes_a87	87,89028	50,30417	2319	treeless vegetation	Tundra/Dryas	Altai alpine meadow and tundra	Montane Grasslands & Shrublands
FPD3	36760	Kunes_a59	87,64611	50,30750	1342	treeless vegetation	Saline grassland	Altai alpine meadow and tundra	Montane Grasslands & Shrublands
FPD3	36917	Kunes_a59	87,64611	50,30750	1342	treeless vegetation	Saline grassland	Altai alpine meadow and tundra	Montane Grasslands & Shrublands
FPD3	37153	Kunes_a87	87,89028	50,30417	2319	treeless vegetation	Tundra/Dryas	Altai alpine meadow and tundra	Montane Grasslands & Shrublands
FPD3	37231	Kunes_a43	87,80472	50,11472	2013	closed forest	Hemiboreal forest/Pinus sibirica-Larix	Altai alpine meadow and tundra	Montane Grasslands & Shrublands
FPD3	37310	Kunes_a87	87,89028	50,30417	2319	treeless vegetation	Tundra/Dryas	Altai alpine meadow and tundra	Montane Grasslands & Shrublands
FPD3	37389	Ryabogina_a27	65,54812	56,48371	64	wetland	near-water vegetation (reeds, sedges, duckweed)	Western Siberian hemiboreal forests	Temperate Broadleaf & Mixed Forests
FPD3	37467	Kunes_a87	87,89028	50,30417	2319	treeless vegetation	Tundra/Dryas	Altai alpine meadow and tundra	Montane Grasslands & Shrublands
FPD3	37546	EPDcoretop_E1193	11,67833	46,76083	870	wetland bog	Typha, Phragmites, Utricularia	Alps conifer and mixed forests	Temperate Conifer Forests
FPD3	37624	EPDcoretop_E320	60,75000	56,76667	230	wetland bog	Pinus sylvestris, Betula pubescens	West Siberian taiga	Boreal Forests/Taiga

FPD3	37702	Ryabogina_a27	65,54812	56,48371	64	wetland	near-water vegetation (reeds, sedges, duckweed)	Western Siberian hemiboreal forests	Temperate Broadleaf & Mixed Forests
FPD3	37780	Kunes_a87	87,89028	50,30417	2319	treeless vegetation	Tundra/Dryas	Altai alpine meadow and tundra	Montane Grasslands & Shrublands
FPD3	37858	Ryabogina_a27	65,54812	56,48371	64	wetland	near-water vegetation (reeds, sedges, duckweed)	Western Siberian hemiboreal forests	Temperate Broadleaf & Mixed Forests
FPD3	37937	Ryabogina_a27	65,54812	56,48371	64	wetland	near-water vegetation (reeds, sedges, duckweed)	Western Siberian hemiboreal forests	Temperate Broadleaf & Mixed Forests
FPD3	38015	Ryabogina_a27	65,54812	56,48371	64	wetland	near-water vegetation (reeds, sedges, duckweed)	Western Siberian hemiboreal forests	Temperate Broadleaf & Mixed Forests
FPD3	38093	Kunes_a87	87,89028	50,30417	2319	treeless vegetation	Tundra/Dryas	Altai alpine meadow and tundra	Montane Grasslands & Shrublands
FPD3	38249	Kunes_a59	87,64611	50,30750	1342	treeless vegetation	Saline grassland	Altai alpine meadow and tundra	Montane Grasslands & Shrublands
FPD3	38406	Kunes_a43	87,80472	50,11472	2013	closed forest	Hemiboreal forest/Pinus sibirica-Larix	Altai alpine meadow and tundra	Montane Grasslands & Shrublands
FPD3	38562	Kunes_a59	87,64611	50,30750	1342	treeless vegetation	Saline grassland	Altai alpine meadow and tundra	Montane Grasslands & Shrublands
FPD3	38670	Kunes_a59	87,64611	50,30750	1342	treeless vegetation	Saline grassland	Altai alpine meadow and tundra	Montane Grasslands & Shrublands

FPD3	38707	Ryabogina_a31	66,12862	56,44082	58	open forest	Grass-meadow grass, there are traces of grazing - plantain, goosefoot, wormwood. Woods with birch, an admixture of pine	Western Siberian hemiboreal forests	Temperate Broadleaf & Mixed Forests
FPD3	38781	Kunes_a87	87,89028	50,30417	2319	treeless vegetation	Tundra/Dryas	Altai alpine meadow and tundra	Montane Grasslands & Shrublands
FPD3	38854	Kunes_a107	91,16028	53,03694	806	treeless vegetation	Meadow steppe	South Siberian forest steppe	Temperate Grasslands, Savannas & Shrublands
FPD3	38926	Ryabogina_a27	65,54812	56,48371	64	wetland	near-water vegetation (reeds, sedges, duckweed)	Western Siberian hemiboreal forests	Temperate Broadleaf & Mixed Forests
FPD3	38999	Tonkov_o1	23,40618	41,74560	2230	treeless vegetation	Groups of Pinus mugo within patches of herb vegetation	Rodope montane mixed forests	Temperate Broadleaf & Mixed Forests
FPD3	39072	Tonkov_o1	23,40618	41,74560	2230	treeless vegetation	Groups of Pinus mugo within patches of herb vegetation	Rodope montane mixed forests	Temperate Broadleaf & Mixed Forests
FPD3	39145	Lapteva_b86	59,03333	54,10000	477	natural grassland	steppe. LocalVeg: herb-	Kazakh forest steppe	Temperate Grasslands,

							bunchgrass steppe		Savannas & Shrublands
FPD3	39217	Novenko_b6	37,62833	54,04417	242	closed forest	broad-leaved forest	East European forest steppe	Temperate Broadleaf & Mixed Forests
FPD3	39254	Dambach_a269	8,17640	49,02110	130	0	0	Western European broadleaf forests	Temperate Broadleaf & Mixed Forests
FPD3	39326	Tonkov_o1	23,40618	41,74560	2230	treeless vegetation	Groups of Pinus mugo within patches of herb vegetation	Rodope montane mixed forests	Temperate Broadleaf & Mixed Forests
**FPD3**	**39363**	**Dambach_a279**	**8,16280**	**49,02650**	**140**	**0**	**0**	**Western European broadleaf forests**	**Temperate Broadleaf & Mixed Forests**
FPD3	39400	Ryabogina_a8	67,03955	57,39380	59	closed forest	Pine forest, cowberry	Western Siberian hemiboreal forests	Temperate Broadleaf & Mixed Forests
FPD2	39437	Dambach_a279	8,16280	49,02650	140	0	0	Western European broadleaf forests	Temperate Broadleaf & Mixed Forests
FPD2	39473	Dambach_a369	7,93520	49,66130	415	0	0	Western European broadleaf forests	Temperate Broadleaf & Mixed Forests
**FPD2**	**39510**	**Dambach_a369**	**7,93520**	**49,66130**	**415**	**0**	**0**	**Western European broadleaf forests**	**Temperate Broadleaf & Mixed Forests**
**FPD2**	**39547**	**Dambach_a369**	**7,93520**	**49,66130**	**415**	**0**	**0**	**Western European broadleaf forests**	**Temperate Broadleaf & Mixed Forests**
**FPD2**	**39583**	**Dambach_a276**	**8,16280**	**49,02650**	**140**	**0**	**0**	**Western European broadleaf forests**	**Temperate Broadleaf & Mixed Forests**

**FPD2**	**39620**	**Dambach_a369**	**7,93520**	**49,66130**	**415**	**0**	**0**	**Western European broadleaf forests**	**Temperate Broadleaf & Mixed Forests**
**FPD2**	**39656**	**Dambach_a276**	**8,16280**	**49,02650**	**140**	**0**	**0**	**Western European broadleaf forests**	**Temperate Broadleaf & Mixed Forests**
**FPD2**	**39692**	**Dambach_a279**	**8,16280**	**49,02650**	**140**	**0**	**0**	**Western European broadleaf forests**	**Temperate Broadleaf & Mixed Forests**
**FPD2**	**39729**	**Dambach_a369**	**7,93520**	**49,66130**	**415**	**0**	**0**	**Western European broadleaf forests**	**Temperate Broadleaf & Mixed Forests**
**FPD2**	**39766**	**Dambach_a369**	**7,93520**	**49,66130**	**415**	**0**	**0**	**Western European broadleaf forests**	**Temperate Broadleaf & Mixed Forests**
**FPD2**	**39802**	**Dambach_a369**	**7,93520**	**49,66130**	**415**	**0**	**0**	**Western European broadleaf forests**	**Temperate Broadleaf & Mixed Forests**
FPD2	39838	Dambach_a369	7,93520	49,66130	415	0	0	Western European broadleaf forests	Temperate Broadleaf & Mixed Forests
**FPD2**	**39874**	**Dambach_a369**	**7,93520**	**49,66130**	**415**	**0**	**0**	**Western European broadleaf forests**	**Temperate Broadleaf & Mixed Forests**
**FPD2**	**39911**	**Dambach_a279**	**8,16280**	**49,02650**	**140**	**0**	**0**	**Western European broadleaf forests**	**Temperate Broadleaf & Mixed Forests**
FPD2	39948	Dambach_a368	7,93520	49,66130	415	0	0	Western European broadleaf forests	Temperate Broadleaf & Mixed Forests
FPD2	39984	Dambach_a279	8,16280	49,02650	140	0	0	Western European broadleaf forests	Temperate Broadleaf & Mixed Forests

FPD2	40021	Novenko_c7	38,59060	53,67060	155	wetland bog	eutrophic peatland on floodplain	East European forest steppe	Temperate Broadleaf & Mixed Forests
FPD2	40094	Dambach_a269	8,17640	49,02110	130	0	0	Western European broadleaf forests	Temperate Broadleaf & Mixed Forests
**FPD2**	**40166**	**Dambach_a277**	**8,16280**	**49,02650**	**140**	**0**	**0**	**Western European broadleaf forests**	**Temperate Broadleaf & Mixed Forests**
**FPD2**	**40238**	**Dambach_a277**	**8,16280**	**49,02650**	**140**	**0**	**0**	**Western European broadleaf forests**	**Temperate Broadleaf & Mixed Forests**
FPD2	40311	Novenko_b6	37,62833	54,04417	242	closed forest	broad-leaved forest	East European forest steppe	Temperate Broadleaf & Mixed Forests
FPD2	40385	Kunes_a137	92,21306	52,24278	2013	treeless vegetation	Alpine grassland	Sayan montane conifer forests	Temperate Conifer Forests
**FPD2**	**40458**	**Kunes_a59**	**87,64611**	**50,30750**	**1342**	**treeless vegetation**	**Saline grassland**	**Altai alpine meadow and tundra**	**Montane Grasslands & Shrublands**
**FPD2**	**40531**	**EPDcoretop_E2005**	**0,13000**	**47,28000**	**28**	**riverine**	**cultivated fields, poplar**	**European Atlantic mixed forests**	**Temperate Broadleaf & Mixed Forests**
**FPD2**	**40604**	**Dambach_a279**	**8,16280**	**49,02650**	**140**	**0**	**0**	**Western European broadleaf forests**	**Temperate Broadleaf & Mixed Forests**
FPD2	40678	Dambach_a277	8,16280	49,02650	140	0	0	Western European broadleaf forests	Temperate Broadleaf & Mixed Forests
**FPD2**	**40750**	**Lapteva_a60**	**57,88907**	**54,89668**	**451**	**0**	**forest-steppe**	**Urals montane forest and taiga**	**Boreal Forests/Taiga**
**FPD2**	**40822**	**Dambach_a277**	**8,16280**	**49,02650**	**140**	**0**	**0**	**Western European broadleaf forests**	**Temperate Broadleaf & Mixed Forests**

FPD2	40895	Novenko_b4	38,60961	53,67365	176	pasture	Northern forest steppe	East European forest steppe	Temperate Broadleaf & Mixed Forests
FPD2	40968	Novenko_b4	38,60961	53,67365	176	pasture	Northern forest steppe	East European forest steppe	Temperate Broadleaf & Mixed Forests
**FPD2**	**41041**	**Novenko_c5**	**38,70250**	**53,63944**	**215**	**fallow**	**abandoned cropland**	**East European forest steppe**	**Temperate Broadleaf & Mixed Forests**
**FPD2**	**41187**	**Dambach_a369**	**7,93520**	**49,66130**	**415**	**0**	**0**	**Western European broadleaf forests**	**Temperate Broadleaf & Mixed Forests**
**FPD2**	**41260**	**Novenko_b4**	**38,60961**	**53,67365**	**176**	**pasture**	**Northern forest steppe**	**East European forest steppe**	**Temperate Broadleaf & Mixed Forests**
FPD2	41332	EPDcoretop_E647	21,61667	49,70000	220	ephemeral lake/pond	fields and meadows	Carpathian montane forests	Temperate Conifer Forests
FPD2	41480	Lapteva_b5	60,84237	56,25898	295	closed forest	coniferous subtaiga. LocalVeg: pine forest	West Siberian taiga	Boreal Forests/Taiga
FPD2	41552	Novenko_c80	32,97980	56,97980	255	fallow	meadow near forest	Sarmatic mixed forests	Temperate Broadleaf & Mixed Forests
FPD2	41624	Novenko_c5	38,70250	53,63944	215	fallow	abandoned cropland	East European forest steppe	Temperate Broadleaf & Mixed Forests
FPD2	41696	Tonkov_o1	23,40618	41,74560	2230	treeless vegetation	Groups of Pinus mugo within patches of herb vegetation	Rodope montane mixed forests	Temperate Broadleaf & Mixed Forests

FPD2	41769	Dambach_a369	7,93520	49,66130	415	0	0	Western European broadleaf forests	Temperate Broadleaf & Mixed Forests
FPD2	41842	Novenko_c80	32,97980	56,97980	255	fallow	meadow near forest	Sarmatic mixed forests	Temperate Broadleaf & Mixed Forests
FPD2	41915	EPDcoretop_E647	21,61667	49,70000	220	ephemeral lake/pond	fields and meadows	Carpathian montane forests	Temperate Conifer Forests
FPD2	41988	Lapteva_a60	57,88907	54,89668	451	0	forest-steppe	Urals montane forest and taiga	Boreal Forests/Taiga
FPD2	42061	Tonkov_o1	23,40618	41,74560	2230	treeless vegetation	Groups of Pinus mugo within patches of herb vegetation	Rodope montane mixed forests	Temperate Broadleaf & Mixed Forests
FPD1	42134	Novenko_c80	32,97980	56,97980	255	fallow	meadow near forest	Sarmatic mixed forests	Temperate Broadleaf & Mixed Forests
FPD1	42338	Dambach_a18	15,38330	48,38330	790	0	0	Central European mixed forests	Temperate Broadleaf & Mixed Forests
FPD1	42543	Novenko_c80	32,97980	56,97980	255	fallow	meadow near forest	Sarmatic mixed forests	Temperate Broadleaf & Mixed Forests
**FPD1**	**42748**	**Novenko_b4**	**38,60961**	**53,67365**	**176**	**pasture**	**Northern forest steppe**	**East European forest steppe**	**Temperate Broadleaf & Mixed Forests**
FPD1	42951	Novenko_c80	32,97980	56,97980	255	fallow	meadow near forest	Sarmatic mixed forests	Temperate Broadleaf & Mixed Forests
**FPD1**	**43155**	**Pidek_a19**	**23,05778**	**50,59472**	**257**	**open forest**	**Nemoral mixed forest, with Picea,**	**Central European mixed forests**	**Temperate Broadleaf & Mixed Forests**

							**Abies, Fagus - national park**		
FPD1	43359	Novenko_c80	32,97980	56,97980	255	fallow	meadow near forest	Sarmatic mixed forests	Temperate Broadleaf & Mixed Forests
FPD1	43563	Novenko_c80	32,97980	56,97980	255	fallow	meadow near forest	Sarmatic mixed forests	Temperate Broadleaf & Mixed Forests
FPD1	43769	Lapteva_b5	60,84237	56,25898	295	closed forest	coniferous subtaiga. LocalVeg: pine forest	West Siberian taiga	Boreal Forests/Taiga
FPD1	43972	Kunes_a87	87,89028	50,30417	2319	treeless vegetation	Tundra/Dryas	Altai alpine meadow and tundra	Montane Grasslands & Shrublands
FPD1	44175	Novenko_c80	32,97980	56,97980	255	fallow	meadow near forest	Sarmatic mixed forests	Temperate Broadleaf & Mixed Forests
FPD1	44380	Pidek_a6	23,06694	50,58222	305	pasture	Nemoral mixed forest, with Picea, Abies, Fagus - national park	Central European mixed forests	Temperate Broadleaf & Mixed Forests
FPD1	44584	Novenko_c78	32,97640	56,45420	246	fallow	meadow near the village	Sarmatic mixed forests	Temperate Broadleaf & Mixed Forests
FPD1	44890	Blyakharchuk_a16	89,34345	50,22395	2748	treeless vegetation	High-mountain area Dzhulukul. Herb—sedge-grass steppe near high-mountain lake.	Sayan alpine meadows and tundra	Montane Grasslands & Shrublands

**FPD1**	**45091**	**Dambach_a279**	**8,16280**	**49,02650**	**140**	**0**	**0**	**Western European broadleaf forests**	**Temperate Broadleaf & Mixed Forests**
FPD1	45398	Kunes_a137	92,21306	52,24278	2013	treeless vegetation	Alpine grassland	Sayan montane conifer forests	Temperate Conifer Forests
FPD1	45804	Pidek_a19	23,05778	50,59472	257	open forest	Nemoral mixed forest, with Picea, Abies, Fagus - national park	Central European mixed forests	Temperate Broadleaf & Mixed Forests
FPD1	46315	Kunes_a137	92,21306	52,24278	2013	treeless vegetation	Alpine grassland	Sayan montane conifer forests	Temperate Conifer Forests
FPD1	46723	Pidek_a19	23,05778	50,59472	257	open forest	Nemoral mixed forest, with Picea, Abies, Fagus - national park	Central European mixed forests	Temperate Broadleaf & Mixed Forests
FPD1	47027	Kunes_a137	92,21306	52,24278	2013	treeless vegetation	Alpine grassland	Sayan montane conifer forests	Temperate Conifer Forests
FPD1	47232	Kolaczek_i30	22,44000	54,33000	187	closed forest	LV: Picea abies, Sphagnum sp., Vaccinium myrtillus	Central European mixed forests	Temperate Broadleaf & Mixed Forests
FPD1	47437	Kunes_a137	92,21306	52,24278	2013	treeless vegetation	Alpine grassland	Sayan montane conifer forests	Temperate Conifer Forests
FPD1	47745	Kunes_a137	92,21306	52,24278	2013	treeless vegetation	Alpine grassland	Sayan montane conifer forests	Temperate Conifer Forests
FPD1	47847	Lapteva_b86	59,03333	54,10000	477	natural grassland	steppe. LocalVeg: herb-bunchgrass steppe	Kazakh forest steppe	Temperate Grasslands, Savannas & Shrublands
FPD1	48051	Kunes_a137	92,21306	52,24278	2013	treeless vegetation	Alpine grassland	Sayan montane conifer forests	Temperate Conifer Forests

FPD1	48457	Kunes_a137	92,21306	52,24278	2013	treeless vegetation	Alpine grassland	Sayan montane conifer forests	Temperate Conifer Forests
FPD1	48865	Lapteva_a53	58,84255	54,52010	1582	forest undefined	boreal forests	Urals montane forest and taiga	Boreal Forests/Taiga
FPD1	49171	Kunes_a137	92,21306	52,24278	2013	treeless vegetation	Alpine grassland	Sayan montane conifer forests	Temperate Conifer Forests
FPD1	49480	Kunes_a137	92,21306	52,24278	2013	treeless vegetation	Alpine grassland	Sayan montane conifer forests	Temperate Conifer Forests
FPD1	49681	Kunes_a137	92,21306	52,24278	2013	treeless vegetation	Alpine grassland	Sayan montane conifer forests	Temperate Conifer Forests
FPD1	49886	Lapteva_b86	59,03333	54,10000	477	natural grassland	steppe. LocalVeg: herb-bunchgrass steppe	Kazakh forest steppe	Temperate Grasslands, Savannas & Shrublands
FPD1	50089	Kunes_a82	87,89028	50,29556	2216	treeless vegetation	Alpine grassland	Altai alpine meadow and tundra	Montane Grasslands & Shrublands
FPD1	50496	Lapteva_a2	61,24602	54,67487	244	0	forest-steppe	Kazakh forest steppe	Temperate Grasslands, Savannas & Shrublands
FPD1	50698	EPDcoretop_E647	21,61667	49,70000	220	ephemeral lake/pond	fields and meadows	Carpathian montane forests	Temperate Conifer Forests
FPD1	50902	Ortu_b45	6,97800	44,68800	2633	0	0	Alps conifer and mixed forests	Temperate Conifer Forests
FPD1	51413	Pidek_a19	23,05778	50,59472	257	open forest	Nemoral mixed forest, with Picea, Abies, Fagus - national park	Central European mixed forests	Temperate Broadleaf & Mixed Forests
FPD1	51928	Lapteva_a2	61,24602	54,67487	244	0	forest-steppe	Kazakh forest steppe	Temperate Grasslands,

									Savannas & Shrublands
**FPD1**	**52437**	**Pidek_a19**	**23,05778**	**50,59472**	**257**	**open forest**	**Nemoral mixed forest, with Picea, Abies, Fagus - national park**	**Central European mixed forests**	**Temperate Broadleaf & Mixed Forests**
**FPD1**	**52945**	**Kunes_a90**	**87,88861**	**50,30583**	**2499**	**treeless vegetation**	**Open scree**	**Altai alpine meadow and tundra**	**Montane Grasslands & Shrublands**
FPD1	53968	Pidek_a6	23,06694	50,58222	305	pasture	Nemoral mixed forest, with Picea, Abies, Fagus - national park	Central European mixed forests	Temperate Broadleaf & Mixed Forests
FPD1	54988	Pidek_a5	23,06306	50,58694	279	closed forest	Nemoral mixed forest, with Picea, Abies, Fagus - national park	Central European mixed forests	Temperate Broadleaf & Mixed Forests
FPD1	55498	Pidek_a19	23,05778	50,59472	257	open forest	Nemoral mixed forest, with Picea, Abies, Fagus - national park	Central European mixed forests	Temperate Broadleaf & Mixed Forests
FPD1	56010	Kolaczek_i30	22,44000	54,33000	187	closed forest	LV: Picea abies, Sphagnum sp., Vaccinium myrtillus	Central European mixed forests	Temperate Broadleaf & Mixed Forests
FPD1	56723	EPDcoretop_E2327	38,59000	53,67000	161	mire	agricultural land	East European forest steppe	Temperate Broadleaf & Mixed Forests

FPD1	57130	Novenko_c45	40,94190	55,13850	93	fallow	abandoned cropland with small pine	Sarmatic mixed forests	Temperate Broadleaf & Mixed Forests
FPD1	57434	Kolaczek_i30	22,44000	54,33000	187	closed forest	LV: Picea abies, Sphagnum sp., Vaccinium myrtillus	Central European mixed forests	Temperate Broadleaf & Mixed Forests
FPD1	58147	EPDcoretop_E375	17,39028	52,55694	109	lake	cultivated fields, deforestated area	Central European mixed forests	Temperate Broadleaf & Mixed Forests
FPD1	58559	Pidek_a6	23,06694	50,58222	305	pasture	Nemoral mixed forest, with Picea, Abies, Fagus - national park	Central European mixed forests	Temperate Broadleaf & Mixed Forests
FPD1	59068	Lapteva_b86	59,03333	54,10000	477	natural grassland	steppe. LocalVeg: herb-bunchgrass steppe	Kazakh forest steppe	Temperate Grasslands, Savannas & Shrublands
FPD1	59580	Lapteva_b106	58,76667	52,91667	329	cave	steppe	Kazakh forest steppe	Temperate Grasslands, Savannas & Shrublands
FPD1	60090	Lapteva_b106	58,76667	52,91667	329	cave	steppe	Kazakh forest steppe	Temperate Grasslands, Savannas & Shrublands

Table S1 - Coordinates and metadata for the best (first) modern analogues of Fimon PD fossil pollen spectra. Low similarity/non analogue situations are highlighted in bold type.

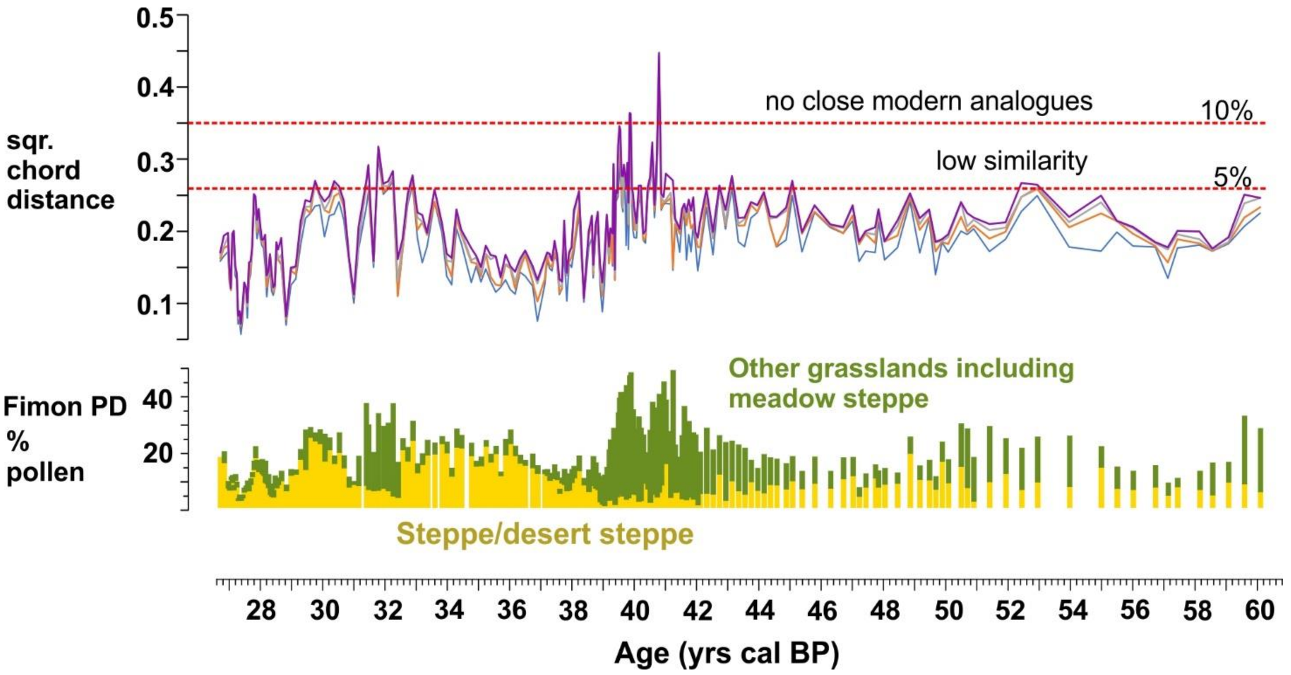

Figure S9 – Comparison between Fimon PD pollen data: sum of grasslands (Cichorioideae, *Anthemis* type, *Aster* type, *Xeranthemum inapertum* type, *Ranunculaceae*, *Geranium molle* type *Ephedra fragilis* type, *Ephedra distachya* type); steppe (*Artemisia*, Chenopodiaceae, *Hippophae*) and squared-chord distance curves for the calibration set, distances smaller than the 5th percentile of all distances between the calibration set samples are "good analogues", while distances larger than the 10th percentile are "non-analogue" assemblages[46].

References

1. Fontana, A., Mozzi, P. & Bondesan, A. Alluvial megafans in the Venetian-Friulian Plain (north-eastern Italy): Evidence of sedimentary and erosive phases during Late Pleistocene and Holocene. *Quaternary International* **189**, 71–90 (2008).

2. Massironi, M., Zampieri, D. & Caporali, A. Miocene to present major fault linkages through the Adriatic indenter and the Austroalpine-Penninic collisional wedge (Alps of NE Italy). *Geological Society Special Publication* **262**, 245–258 (2006).

3. Pola, M., Ricciato, A., Fantoni, R., Fabbri, P. & Zampieri, D. Architecture of the western margin of the North Adriatic foreland: the Schio-Vicenza fault system. *Italian Journal of Geoscience* **133**, 223–234 (2014).

4. Macera, P. *et al.* Geodynamic implications of deep mantle upwelling in the source of Tertiary volcanics from the Veneto region (South-Eastern Alps). *Journal of Geodynamics* **36**, 563–590 (2003).

5. Cavinato, A. L'eolico nei M. Berici e Lessini. *Accademia nazionale dei Lincei* (1963).

6. Pini, R., Ravazzi, C. & Reimer, P. J. The vegetation and climate history of the last glacial cycle in a new pollen record from Lake Fimon (southern Alpine foreland, N-Italy). *Quaternary Science Reviews* **29**, 3115–3137 (2010).

7. Monegato, G., Pini, R., Ravazzi, C., Reimer, P. J. & Wick, L. Correlating Alpine glaciation with Adriatic sea-level changes through lake and alluvial stratigraphy. *J Quat Sci* **26**, 791–804 (2011).

8. Badino, F. *et al.* The fast-acting "pulse" of Heinrich Stadial 3 in a mid-latitude boreal ecosystem. *Sci Rep* **10**, 1–14 (2020).

9. Monegato, G., Pini, R., Ravazzi, C., Reimer, P. J. & Wick, L. Correlating Alpine glaciation with Adriatic sea-level changes through lake and alluvial stratigraphy. *Journal of Quaternary Science* **26**, 791–804 (2011).

10. Pini, R. *et al.* Linking North Atlantic and Alpine Last Glacial Maximum climates via a high-resolution pollen-based subarctic forest steppe record. *Quat Sci Rev* **294**, (2022).

11. Abu Zeid, N., Bignardi, S., Russo, P. & Peresani, M. Deep in a Paleolithic archive: Integrated geophysical investigations and laser-scanner reconstruction at Fumane Cave, Italy. *Journal of Archaeological Science: Reports* **27**, 101976 (2019).

12. Peresani, M. *et al.* Age of the final Middle Palaeolithic and Uluzzian levels at Fumane Cave, Northern Italy, using 14C, ESR, 234U/230Th and thermoluminescence methods. *Journal of Archaeological Science* **35**, 2986–2996 (2008).

13. Peresani, M. Fifty thousand years of flint knapping and tool shaping across the Mousterian and Uluzzian sequence of Fumane cave. *Quaternary International* **247**, 125–150 (2012).

14. Peresani, M., Cristiani, E. & Romandini, M. The Uluzzian technology of Grotta di Fumane and its implication for reconstructing cultural dynamics in the Middle-Upper Palaeolithic transition of Western Eurasia. *J Hum Evol* **91**, 36–56 (2016).

15. Falcucci, A., Conard, N. J. & Peresani, M. *A critical assessment of the Protoaurignacian lithic technology at Fumane Cave and its implications for the definition of the earliest Aurignacian*. *PLoS ONE* vol. 12 (2017).

16. Falcucci, A., Conard, N. J. & Peresani, M. Breaking through the aquitaine frame: A re-evaluation on the significance of regional variants during the Aurignacian as seen from a key record in southern Europe. *Journal of Anthropological Sciences* **98**, 99–140 (2020).

17. Falcucci, A. & Peresani, M. A pre-Heinrich Event 3 assemblage at Fumane Cave and its contribution for understanding the beginning of the Gravettian in Italy: Ein vor das Heinrich 3. *Qartär* **66**, 135–154 (2019).

18. Sauro, U. The Monti Berici: A Peculiar Type of Karst in the Southern Alps. *Acta Carsologica* **31**, 99–114 (2016).

19. Romandini, M., Bertola, S. & Nannini, N. Nuovi dati sul Paleolitico dei Colli Berici : risultati preliminari dello studio archeozoologico e delle materie prime litiche della Grotta del Buso Doppio del Broion (Lumignano, Longare, Vicenza). in *Preistoria e protostoria del Veneto. - (Studi di preistoria e protostoria ; 2)* 53–59 (2015).

20. De Stefani, M., Gurioli, F. & Ziggiotti, S. Il Paleolitico superiore del Riparo del Broion nei Colli Berici (Vicenza). *Rivista di scienze preistoriche : LV, Supplemento 1* (2005).

21. Peresani, M., Bertola, S., Delpiano, D., Benazzi, S. & Romandini, M. The Uluzzian in the north of Italy: insights around the new evidence at Riparo Broion. *Archaeol Anthropol Sci* **11**, 3503–3536 (2019).

22. Romandini, M. *et al.* A late Neanderthal tooth from northeastern Italy. *J Hum Evol* **147**, 102867 (2020).

23. Leonardi, P. & Broglio, A. Le Paléolithique de la Vénetie. *Annali Università di Ferrara* **15**, 1–118 (1962).

24. Leonardi, P. Risultati della prima campagna di scavo della stazione musteriana di S. Bernardino nei Colli Berici (VI). *Atti Istituto Veneto SS.LL.AA.* **117**, 387–402 (1959).

25. Peresani, M. Sistemi tecnici di produzione litica nel Musteriano d'Italia. Studio tecnologico degli insiemi litici delle unità VI e II della Grotta di San Bernardino (Colli Berici, Veneto). *Rivista Scienze Preistoriche* **47**, 79–167 (1996).

26. Terlato, G. *et al.* Late Neanderthal Subsistence At San Bernardino Cave (Berici Hills - Northeastern Italy) Inferred From Zooarchaeological Data. *Alpine and Mediterranean Quaternary* **34**, 213–235 (2021).

27. Fiore, I., Gala, M. & Tagliacozzo, A. Ecology and subsistence strategies in the Eastern Italian Alps during the Middle Palaeolithic. *International Journal of Osteoarchaeology* **14**, 273–286 (2004).

28. Romandini, M. *et al.* Macromammal and bird assemblages across the late Middle to Upper Palaeolithic transition in Italy: an extended zooarchaeological review. *Quaternary International* **551**, (2020).

29. Cassoli, P. F. & Tagliacozzo, A. I resti ossei di macromammiferi, uccelli e pesci della Grotta maggiore di San Bernardino sui Colli Berici (VI): considerazioni paleoeconomiche, paleoecologiche e cronologiche. *Bull. di Paletnol. Ital* **85**, 1–71 (1994).

30. Cassoli, P. F. & Tagliacozzo, A. Considerazioni paleontologiche, paleoecologiche e archeozoologiche sui macromammiferi e gli uccelli dei livelli del Pleistocene superiore del Riparo di Fumane (VR) scavi 1988-91. *Boll. Museo Civico di Storia Naturale di Verona* **23**, 85–117 (1994).

31. Terlato, G. *et al.* Chronological and Isotopic data support a revision for the timing of cave bear extinction in Mediterranean Europe. *Historical Biology* **31**, 474–484 (2019).

32. Gallini, V. & Sala, B. Settepolesini di Bondeno (Ferrara - Eastern Po Valley): the first example of mammoth steppe in Italy. *The World of Elephants - International Congress* 272–275 (2001).

33. Romandini, M. *et al.* Bears and humans, a Neanderthal tale. Reconstructing uncommon behaviors from zooarchaeological evidence in southern Europe. *Journal of Archaeological Science* **90**, 71–91 (2018).

34. Gurioli, F., Cappato, N., De Stefani, M. & Tagliacozzo, A. Considerazioni Paleontologiche, Paleoecologiche e Archeozoologiche dei livelli del Paleolitico superiore del Riparo del Broion (Colli Berici, Vicenza). *Atti del V Convegno Nazionale di Archeozoologia* 47–56 (2006).

35. Carrera, L., Pavia, M., Peresani, M. & Romandini, M. Late Pleistocene fossil birds from Buso Doppio del Broion Cave (North-Eastern Italy): implications for palaeoecology, palaeoenvironment and palaeoclimate. *Bollettino della Società Paleontologica Italiana* **57**, 145–174 (2018).

36. Nannini, N. & Romandini, M. Gravettian and Epigravettian hunters in the Berici Hills (Vicenza): two case of cave bear (Ursus spelaeus) exploitation. Cacciatori gravettiani ed epigravettiani nei Colli Berici (Vicenza): due casi di sfruttamento dell'orso delle caverne (Ursus spelaeus). *Atti del 7° Convegno Nazionale di Archeozoologia a cura di U. Thun Hohenstein* **11**, (2015).

37. Pini, R. *et al.* Linking North Atlantic and Alpine Last Glacial Maximum climates via a high-resolution pollen-based subarctic forest steppe record. *Quat Sci Rev* **294**, (2022).

38. Gretzinger, J. *et al.* Large-scale mitogenomic analysis of the phylogeography of the Late Pleistocene cave bear. *Sci Rep* **9**, (2019).

39. Terlato, G. *et al.* Extinction and paleoecology of the late pleistocene cave bear from northeastern Italy: Radiocarbon and stable isotope evidence. *Alpine and Mediterranean Quaternary* **31**, 99–103 (2018).

40. Carrera, L. Birds from the late pleistocene: Environmental and climatic scenarios between the Alps and the Great Adriatic Plain (North-Eastern Italy). *Alpine and Mediterranean Quaternary* **31**, 49–53 (2018).

41. Pini, R., Ravazzi, C. & Reimer, P. J. The vegetation and climate history of the last glacial cycle in a new pollen record from Lake Fimon (southern Alpine foreland, N-Italy). *Quat Sci Rev* **29**, 3115–3137 (2010).

42. Bronk Ramsey, C. Bayesian Analysis of Radiocarbon Dates. *Radiocarbon* **51**, 337–360 (2009).

43. Reimer, P. *et al.* The IntCal20 Northern Hemisphere radiocarbon age calibration curve (0-55 kcal BP). *Radiocarbon* 1–33 (2020) doi:doi:10.1017/RDC.2020.41.

44. Higuera, P. CharAnalysis 0.9 : Diagnostic and analytical tools for sediment-charcoal analysis. *Montana State University, Bozeman, MT* 1–27 Preprint at (2009).

45. Kelly, R. F., Higuera, P. E., Barrett, C. M. & Sheng Hu, F. A signal-to-noise index to quantify the potential for peak detection in sediment-charcoal records. (2011) doi:10.1016/j.yqres.2010.07.011.

46. Simpson, G. L. Analogue Methods in Palaeolimnology. in *Tracking Environmental Change Using Lake Sediments. Developments in Paleoenvironmental Research* (Springer, 2012).
